# Resting Energy Expenditure Prediction Equations in the Pediatric Population: A Systematic Review

**DOI:** 10.3389/fped.2021.795364

**Published:** 2021-12-06

**Authors:** Jimena Fuentes-Servín, Azalia Avila-Nava, Luis E. González-Salazar, Oscar A. Pérez-González, María Del Carmen Servín-Rodas, Aurora E. Serralde-Zuñiga, Isabel Medina-Vera, Martha Guevara-Cruz

**Affiliations:** ^1^Departamento de Metodología de la Investigación, Instituto Nacional de Pediatría, Ciudad de México, Mexico; ^2^Hospital Regional de Alta Especialidad Península de Yucatán, Mérida, Mexico; ^3^Servicio de Nutrición Clínica, Instituto Nacional de Nutrición y Ciencias Médicas Salvador Zubirán, Ciudad de México, Mexico; ^4^Sección de estudios de Posgrado e Investigación, Escuela Superior de Medicina, Instituto Politécnico Nacional, Ciudad de México, Mexico; ^5^Laboratorio de Oncología Experimental, Instituto Nacional de Pediatría, Ciudad de México, Mexico; ^6^Escuela Nacional de Enfermería y Obstetricia, Universidad Nacional Autónoma de México, Ciudad de México, Mexico; ^7^Tecnologico de Monterrey, Escuela de Medicina y Ciencias de la Salud, Ciudad de México, Mexico; ^8^Departamento de Fisiología de la Nutrición, Instituto Nacional de Nutrición y Ciencias Médicas Salvador Zubirán, Ciudad de México, Mexico

**Keywords:** energy expenditure, children, adolescents, indirect calorimetry, predictive equation

## Abstract

**Background and Aims:** The determination of energy requirements is necessary to promote adequate growth and nutritional status in pediatric populations. Currently, several predictive equations have been designed and modified to estimate energy expenditure at rest. Our objectives were (1) to identify the equations designed for energy expenditure prediction and (2) to identify the anthropometric and demographic variables used in the design of the equations for pediatric patients who are healthy and have illness.

**Methods:** A systematic search in the Medline/PubMed, EMBASE and LILACS databases for observational studies published up to January 2021 that reported the design of predictive equations to estimate basal or resting energy expenditure in pediatric populations was carried out. Studies were excluded if the study population included athletes, adult patients, or any patients taking medications that altered energy expenditure. Risk of bias was assessed using the Quality Assessment Tool for Observational Cohort and Cross-Sectional Studies.

**Results:** Of the 769 studies identified in the search, 39 met the inclusion criteria and were analyzed. Predictive equations were established for three pediatric populations: those who were healthy (*n* = 8), those who had overweight or obesity (*n* = 17), and those with a specific clinical situation (*n* = 14). In the healthy pediatric population, the FAO/WHO and Schofield equations had the highest *R*^2^ values, while in the population with obesity, the Molnár and Dietz equations had the highest *R*^2^ values for both boys and girls.

**Conclusions:** Many different predictive equations for energy expenditure in pediatric patients have been published. This review is a compendium of most of these equations; this information will enable clinicians to critically evaluate their use in clinical practice.

**Systematic Review Registration:** https://www.crd.york.ac.uk/prospero/display_record.php?RecordID=226270, PROSPERO [CRD42021226270].

## Introduction

Energy is vital and necessary to maintain the metabolic functions of an organism. The determination of energy requirements in children and adolescents is important for their proper growth as well as for the prevention of the effects of overfeeding or underfeeding. To establish such energy requirements, it is necessary to determine the total energy expenditure (TEE), which is the amount of energy used daily by the individual (1). The largest component of TEE (60–70%) is basal energy expenditure (BEE), which represents the integration of the minimal activity of all body tissues under steady state conditions (2). Methods to determine TEE are not very accessible, and they are expensive. Therefore, the estimation of TEE from total BEE, food thermogenesis and physical activity is very common. Because techniques to measure BEE are impractical, the estimation of BEE from resting energy expenditure (REE) is the most feasible and commonly used method. A widely used reference method for determining REE is indirect calorimetry (IC) (3); however, the use of this technique is not practical due to its high cost, limited availability, long measurement time and the need for appropriate fasting (4, 5), which is why several predictive equations for energy expenditure have been developed or modified for routine clinical practice over time, with the aim of determining caloric requirements in children and adolescents with various clinical situations. Most of these equations were obtained from heterogeneous groups of children; therefore, the aims of the present review were 1) to identify the equations designed to predict energy expenditure and 2) to identify the anthropometric and demographic variables used in the design of the equations for pediatric patients who are healthy and have illness.

## Methods

A systematic review of the published literature was performed according to the Preferred Reporting Items for Systematic Review and Meta-Analysis (PRISMA) guidelines (6). The protocol was registered in the International Prospective Register of Systematic Reviews (PROSPERO) (5, 7) under reference number CRD42021226270.

### Search Strategy

A comprehensive literature search was conducted independently by two authors (JFS and LGS) in the digital Medline/PubMed, EMABSE and Latin American and Caribbean Health Sciences Literature (LILACS) databases. Articles published through January 2021 were searched. The electronic search was supplemented by manual screening of reference lists of relevant articles to identify possible studies not identified in the electronic search. The population, exposure, comparator, outcome (PECO) strategy was applied in the present systematic review, and the descripts were as follows: pediatric patients (population) who are healthy and have illness, predictive equations for energy expenditure (exposure), the reference standard for measuring energy expenditure (comparator), and Pearson's correlation coefficient (R) value or the coefficient of determination (*R*^2^) value (outcome) (Table 1). The search was performed using the following terms: “energy expenditure” OR “energy Metabolism” OR “resting energy expenditure”, OR “basal metabolic rate [MeSH Major Topic],” “predictive equation,” “indirect calorimetry” and “child” OR “adolescent” OR “pediatrics” [MeSH Terms] NOT “adults” NOT “athletes” with no restrictions on the study design, date or language of publication and limited to humans.

**Table 1:** PECO criteria for study selection.

**Criterion**		**Description**
P	Population	Healthy pediatric patients and those with illness
E	Exposure	Predictive equations for energy expenditure
C	Comparator	The reference standard
O	Outcome	Correlation value and/or coefficient of determination

### Studies Sections and Data Extraction

#### Selection of Studies

After the removal of duplicates, titles and abstracts were independently screened by two authors (JFS and LGS) for eligibility according to the inclusion criteria. Published studies identified in the search were initially assessed considering titles and abstracts. Based on the initial assessment, studies were identified as “excluded” or as “full-text assessment for eligibility.”

#### Selection Criteria

Studies were included if they met the following criteria: 1) the study population included pediatric patients (age 0–18 years), 2) energy expenditure measurement was performed by IC, and 3) equations for BEE and REE were developed for patients with different clinical conditions. Studies were excluded if 1) the study population included patients with thyroid problems or patients taking medications that alter energy expenditure, 2) equations were designed for the pediatric athlete population, 3) equations were designed considering combined adult and pediatric populations, or 4) equations were designed considering a different population than the one being evaluated.

#### Data Extraction

The articles that met the inclusion criteria were reviewed by two investigators, and the data were extracted in a specific format and included variables such as 1) the clinical condition of the population studied; 2) the age range of the population studied; 3) the proposed equations; 4) the *R* and/or the *R*^2^ value reported for the correlation between the new equation and the reference standard used; 5) the method used to determine energy expenditure; 6) the country where the models were developed; and 7) in the case of body composition measurement, the documented method to assess body composition. Any discrepancies were resolved by discussion with a third author.

#### Quality Assessment and Bias

Study validity was assessed independently by two authors (JFS and LGS), with potential disagreements resolved by consensus or consultation with a third author (IMV) using the “Quality Assessment Tool for Observational Cohort and Cross-Sectional Studies” developed jointly by methodologists from the National Heart, Lung and Blood Institute (NHLBI) and Research Triangle Institute International (8). The tools included fourteen items to assess potential flaws in the study methodology, including the following sources of bias: patient selection, performance, attrition and detection, confounding, study power, and other factors. A judgment of “good” indicated a low risk of bias, “fair” indicated that the study was susceptible to some bias considered not sufficient to invalidate its results, and “poor” indicated a significant risk of bias.

## Results

### Study Selection and Characteristics

**Figure 1:**
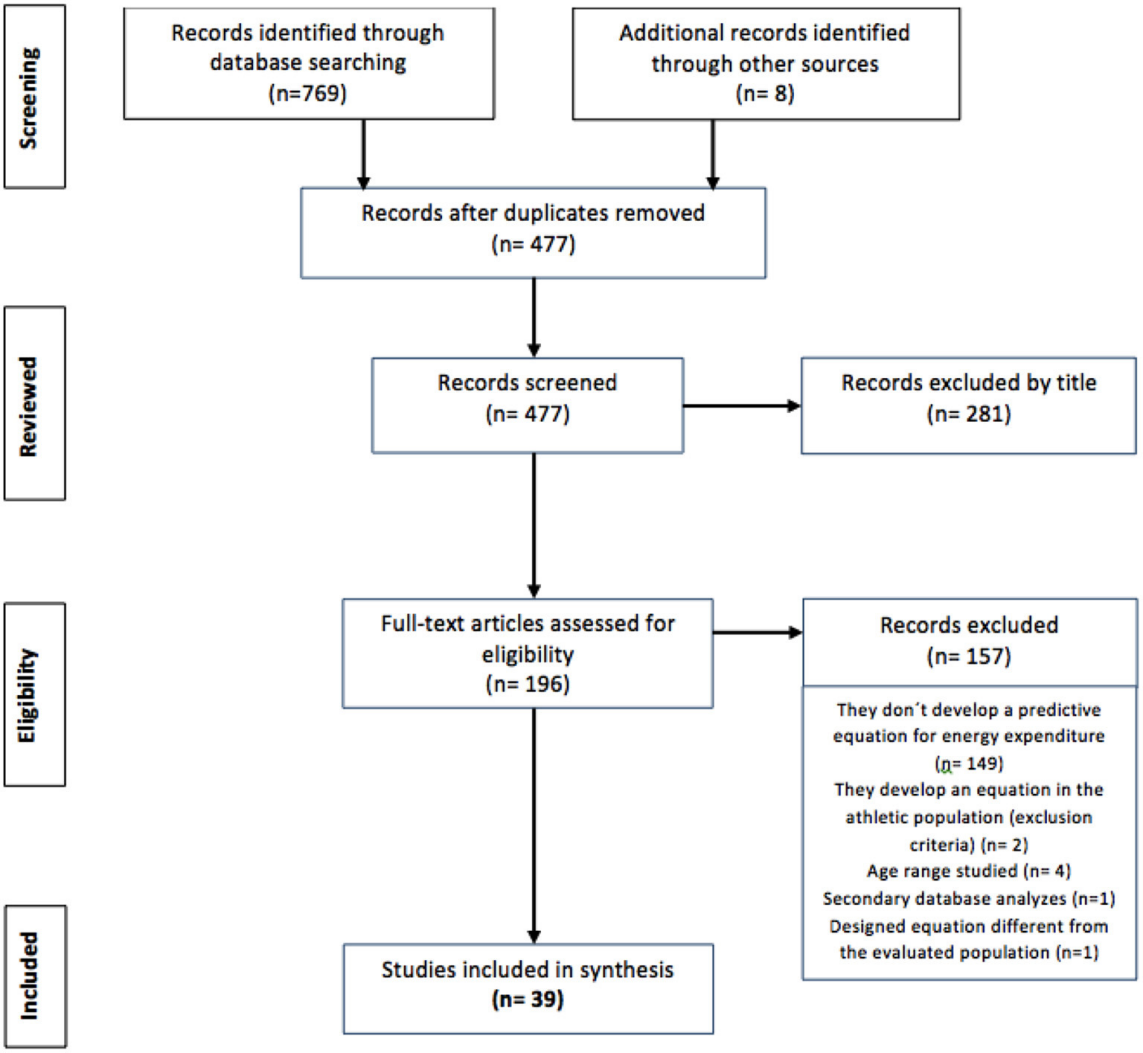
PRISMA flow chart of the included studies.

The search identified 769 possible studies, and among these, 300 were excluded because they were duplicates; therefore, 477 records were identified for possible inclusion in the review. When analyzing the studies on the basis of title and abstract, 281 were eliminated because they did not meet the inclusion criteria. A total of 196 records were retained for full-text assessment, and 157 studies were excluded, the main reason being that the studies did not generate a new predictive equation (*n* = 149), with 2 additional studies being excluded for developing an equation in an athlete population, 4 studies for not studying the appropriate age range, 1 study for not generating an equation but instead providing a secondary analysis of databases and 1 study for designing an equation in a population different from the population of interest in this study. Eight publications were further identified as relevant for this review by cross-referencing and met the selection criteria; therefore, a total of 39 articles were included. However, 40 predictive equations were analyzed because the Institute of Medicine (IOM) article presents equations for healthy populations and populations with obesity (Figure 1).

### Risk of Bias

**Figure 2:**
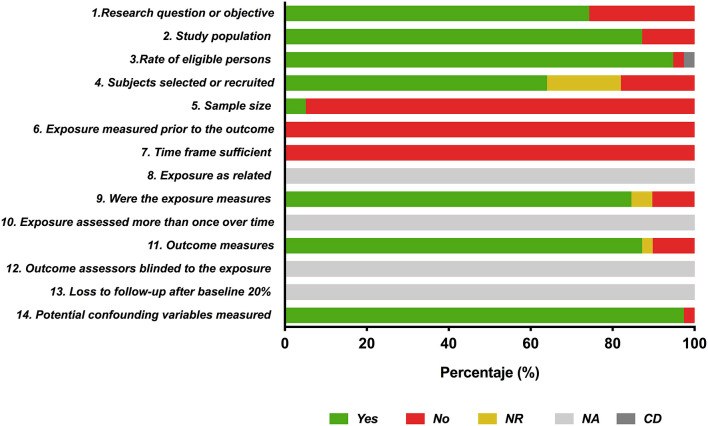
Report of the study quality index components in the included studies. Yes (green bar), No (red bar) NR (yellow bar): not reported; NA (light gray bar): not applicable; CD (dark gray bar): cannot determine.

The quality rating was acceptable, with a moderate risk of bias, in twenty-one of the assessed studies; five studies were rated as poor, with a significant risk of bias, and thirteen studies had an overall good quality rating (Supplementary Figure S1 and Supplementary Table S1). In general, sample size calculation was not documented in the studies (sample size calculation was reported in only one study), and the number of subjects assessed varied across studies. In total, 74.3% of the articles assessed (*n* = 29) reported the generation of a predictive equation in the research question and/or objective (Figure 2).

### Energy Expenditure Prediction Equations

Once the clinical conditions of the populations included the studies were analyzed, equations were stratified into the following three groups to obtain a better understanding of the populations being reviewed: 1) healthy children, who were described as those presenting no significant medical problems; 2) children with overweight or obesity without any other complications; and 3) patients with specific clinical situations (anemia, muscular atrophy type 1, intensive care, surgical procedures and others requiring nutritional therapy). The following is a description of each of the equations in each stratum.

### Prediction Equations in Healthy Pediatric Populations

Eight articles contained predictive equations with reference to a healthy pediatric population, and the predictive equations and their characteristics are presented in Table 2.

**Table 2:** Equations for the prediction of energy expenditure in the healthy pediatric population.

**References**	***N***	**Clinical condition**	**Age range**	**(*R*^*2*^) Predictive equation**	**Calorimeter**	**Notes – Country where it was developed and/or population – Coding of variables in the equation – Body composition assessment method**
Harris and Benedict (7)	136 M 103 W 93 NB	Healthy	21-70 years	***Men*** • h = 66.4730 + (13.7516 *× W*) + (5.0033 *× H*) – (6.7550 *× A*) ***Women*** • h = 655.0955 + (9.5634 *× W*) + (1.8496 *× H*) – (4.6756 *× A*) ***Infants*** • h = 22.104 + (31.049 *× W*) + (1.162 *× H*)	Not reported	• Boston, MA
FAO/WHO/UNU (9)	7,000	Healthy	3–18 years	**Weight** **Boys** **(R**^**2**^ = 0.94)* • **0–3 years** → BEE (kcal/d) = (60.9 *× W*) – 54 (*R*^2^ = 0.73)* • **3–10 years** → BEE (kcal/d) = (22.7 *× W*) + 495 (R^2^ = 0.81)* • **10–18 years** → BEE (kcal/d) = (17.5 *× W*) + 651 **Girls** (R^2^ = 0.94)* • **0–3 years** → BEE (kcal/d) = (61 *× W*) – 51 (R^2^ = 0.72)* • **3–10 years** → BEE (kcal/d) = (22.5 *× W*) + 499 (R^2^ = 0.56)* • **10–18 years** → BEE (kcal/d) = (12.2 *× W*) + 746 **Weight and height** Boys (R^2^ = 0.79)* • **10–18 years** → BEE (kcal/d) = (16.6 *× W*) + (77 *× H*) + 572 Girls (R^2^ = 0.59)* • **10–18 years** → BEE (kcal/d) = (7.4 *× W*) + (482 *× H*) + 217	Not reported	• Developed and underdeveloped countries
Schofield (10)	2,359	Healthy	3–18 years	**Weight** **Boys** (R^2^ = 0.90)* • **0–3 years** → BEE (kcal/d) = (59.51 *× W*) – 30.33 (R^2^ = 0.68)* • **3–10 years** → BEE (kcal/d) = (22.706 *× W*) + 504.3 (R^2^ = 0.86)* • **10–18 years** → BEE (kcal/d) = (13.384 *× W*) + 692.6 **Girls** (R^2^ = 0.92)* • **0–3 years** → BEE (kcal/d) = (58.31 *× W*) – 31.07 (R^2^ = 0.65)* • **3–10 years** → BEE (kcal/d) = (20.315 *× W*) + 485.9 (R^2^ = 0.64)* • **10–18 years** → BEE (kcal/d) = (17.686 *× W*) + 658.2	Not reported	• Italian, American and Asian populations
				**Weight and height** **Boys** (R^2^ = 0.94)* • **0–3 years** → BEE (kcal/d) = (0.167 *× W*) + (1517.4 *× H*) - 617.6 (R^2^ = 0.68)* • **3–10 years** → BEE (kcal/d) = (19.6 *× W*) + (130.3 *× H*) + 414.9 (R^2^ = 0.86)* • **10–18 years** → BEE (kcal/d) = (16.25 *× W*) + (137.2 *× H*) + 515.5 **Girls** (R^2^ = 0.94)* • **0–3 years** → BEE (kcal/d) = (16.25 *× W*) + (1,023.2 *× H*) - 413.5 (R^2^ = 0.65)* • **3–10 years** → BEE (kcal/d) = (16.97 *× W*) + (161.8 *× H*) + 371.2 (R^2^ = 0.67)* • **10–18 years** → BEE (kcal/d) = (8.365 *× W*) + (465 *× H*) + 200		
Henry et al. (11)	78 B 117 G	Healthy	10–15 years	**Equation 1** **Boys** **(R^2^ = 0.61)** • BEE (kJ/d) = (66.9 *× W*) + 2,876 **(R^2^ = 0.62)** • BEE (kJ/d) = (105.4 × FFM) + 0.2230 **(R^2^ = 0.62)** • BEE (kJ/d) = (54.6 *× W*) + (18.8 *× H*) + 0.576 **(R^2^ = 0.63)** • BEE (kJ/d) = (91.1 × FFM) + (29.4 × FM) + 0.2422 **(R^2^ = 0.67)** • BEE (kJ/d) = (78.5 *× W*) + (suprailiac ×45.3 - triceps ×54.99 - subscapular ×38.3) + 294 **Girls** • BEE (kJ/d) = (47.9 *× W*) + 3,230 • BEE (kJ/d) = (21.0 *× W*) – (11.0 *× H*) + (80.7 × FFM) – (154.6 *× A*) + 0.5319 • BEE (kJ/d) = (96.77 × FFM) – (383.9 × G) + (21.4 × FM) – (136.0 *× A*) + 0.3949	Ventilated hood system (Datex Deltatrac, Datex Instrumentation Corp., Helsinki, Finland)	• Oxford, UK • Gender: 1 (boys) or 0 (girls) • Folds in millimeters (mm) • Body composition evaluated by folds
				**Equation 2 (puberty)** **Age of development** **Boys** **(R^2^ = 0.61)** • PH1 → BEE (kJ/d) = (60.0 *× W*) – (194 *× A*) + (50.7 × Wrist breadth) + 2,892 **(R^2^ = 0.69)** • G3 → BEE (kJ/d) = (270 × MUAMC) + log of the sum of 5 skinfolds ×1450) – 1,803		• Wrist breadth in millimeters (mm) • MUAMC: mid-upper-arm muscle Circumference (cm); • **Menarche status:** Premenarche = 0; postmenarche = 1 • PH1: Pubic hair • G3: gonadal development
				**Girls** **(R^2^ = 0.52)** • Breast stage 1 → BEE (kJ/d) = (69.9 *× W*) – 5,230 **(R^2^ = 0.52)** • 10–15 years → BEE (kJ/d) = (50.6 *× W*) – (170.9 × menarche status) + 3,161 **Premenarche** **(R^2^ = 0.89)** • BEE (kJ/d) = (53.6 *× W*) + 3,031 **(R^2^ = 0.75)** • BEE (kJ/d) = (97.07 *× W*) – (74.6 × FM) – (121.2 *× A*) + 3,452		
Institute for Medicine of the National Academies and Food and Nutrition Board (12)	167 B 358 G	Healthy	0–18 years	**Boys (R^2^ = 0.89)** • BEE (kcal/d) = 68 – (43.3 *× A*) + 712 *× H*) + (19.2 *× W*). **Girls (R^2^ = 0.75)** • BEE (kcal/d) = 189 – (17.6 *× A*) + (625 *× H*)) + (7.9 *× W*)	Doubly labeled water technique	• Caucasian, African American, Hispanic, and American Indian populations • Height in meters (m)
Henry (2)	5,794 B 4,702 G	Healthy	3–18 years	**Oxford with weight** **Boys** (R^2^ = 0.910)* • **0–3 years →** BEE (kcal/d) = (61.0 *× W*) – 33.7 (R^2^ = 0.683)* • **3–10 years →** BEE (kcal/d) = (23.3 *× W*) + 514 (R^2^ = 0.741)* • **10–18 years →** BEE (kcal/d) = (18.4 *× W*) + 581 **Girls** (R^2^ = 0.921)* • **0–3 years →** BEE (kcal/d) = (58.9 *× W*) – 23.1 (R^2^ = 0.672)* • **3–10 years →** BEE (kcal/d) = (20.1 *× W*) + 507 (R^2^ = 0.565)* • **10–18 years →** BEE (kcal/d) = (11.1 *× W*) + 761 **Oxford with weight and height** **Boys** (R^2^ = 0.919)* • **0–3 years →** BEE (kcal/d) = (28.2 *× W*) + (859 *× H*) – 371 (R^2^ = 0.697)* • **3–10 years →** BEE (kcal/d) = (15.1 *× W*) + (74.2 *× H*) + 306 (R^2^ = 0.746)* • **10–18 a** → BEE (kcal/d) = (15.6 *× W*) + (266 *× H*) + 299 **Girls** (R^2^ = 0.929)* • **0–3 years →** BEE (kcal/d) = (30.4 *× W*) + (703 *× H*) – 287 (R^2^ = 0.680)* • **3–10 years →** BEE (kcal/d) = (15.9 *× W*) + (210 *× H*) + 349 **(R^2^** **=** 0.574)* • **10–18 years →** BEE (kcal/d) = (9.40 *× W*) + (249 *× H*) + 462	Not reported	• European, American and Asian populations • Known as the Oxford equation • Body composition evaluated by BIA
Lawrence et al. (13)	38 B 54 G	Healthy	4–11 years	**Equation 1 (R^2^ = 0.611)** • REE (kcal/d) = [5.38 × PIBW (%)] + (824.39 × BSA) – (22.47 × BF) - 201.91 **Equation 2 (R^2^ = 0.560)** • REE (kcal/d) = 448.48 + (588.43 × BSA) **Equation 3 (R^2^ = 0.560)** • REE (kcal/d) = 632.40 + (15.66 *× A*) + (9.53 *× W*) **Equation 4 (R^2^ = 0.556)** • REE (kcal/d) = REE = 581.57 + (20.19 × FFM)	TrueOne 2,400 metabolic cart (ModelQMC, ParvoMedics Corp. UT, USA)	• Korean population • Known as Kim equation • PIBW (%) = percent ideal body weight • BSA = body surface area (m^2^) • BSA = (0.007184 × *H*^0.725^ × W ^0.425^)
Kaneko et al. (14)	113 B 108 G	Healthy	6–17 years	**Boys (R^2^ = 0.861)** • REE (kcal/d) = (14.4 *× W*) + (5.09 *× H*) – (34.0 *× A*) + 403 **Girls (R^2^ = 0.628)** • REE (kcal/d) = (7.64 *× W*) + (4.22 *× H*) – (22.5 × A) + 526	Model AR-1, ARCO System Co., Chiba	• Japanese population

*BEE, basal energy expenditure; REE, resting energy expenditure; h, total heat production per 24 h; M, men, W, women; NB, newborns; B, boys; G, girls; W, weight (kg); H, height (cm); A, age (years); FFM, fat-free mass (kg); FM, fat mass (kg); BF, body fat (kg); G, gender (sex), as reported in the original equation; kcal, kilocalories; kJ, kilojoules; BIA, bioelectrical impedance analysis*.

* *To standardize the units, the equations with Pearson's R values were converted by squaring this coefficient to obtain the value of R^2^*.

### Harris-Benedict Equation

The Harris-Benedict equation was developed in 1918 (7) in Boston, MA. In a sample size of 332 healthy individuals [136 males, 103 females and 93 newborns (4 days)], athletes and vegetarians were included to represent the general population. Energy expenditure was determined by IC, and the variables studied were weight, height, pulse and body surface area (BSA). The correlation between height and heat production was documented, and the *R* value for the correlation between body weight and heat production was 0.80 in newborns and men and 0.60 in women. The authors determined that both height and body weight have independent significant effects on the prediction of basal metabolism.

Two equations, which included the variables of weight, height and age, were established to determine 24-h heat production, one for men and one for women. These equations were tabulated for weight values from 25.0 to 124.9 kg, for height values from 151 to 200 cm and for ages from 21 to 70 years. The authors also proposed a second equation for infants, which included the variables of weight in kilograms and height in centimeters, tabulated for weight between 2 and 4 kg and height between 46 and 54 cm.

Neither the *R* value nor the *R*^2^ value of the equations was documented.

### Food and Agriculture Organization/World Health Organization (FAO/WHO) Equation

The FAO/WHO equation (9) was designed in 1985. By compiling the literature (114 studies), a sample size of ~7,000 healthy people of all ages from developed and underdeveloped countries (USA, the UK, India, China, Sweden, Burma, the Netherlands, Brazil, Nepal, Hawaii, Japan, the Philippines, Korea, Jamaica, Mexico, Denmark and Austria) was obtained. A small sample of children with illness was included, which the authors reported was not a significant proportion of the sample. The number of children assessed was not mentioned.

Two different equation models were developed for the prediction of BEE. The first model takes into account the weight variable, is stratified by sex and is further subdivided by age (0–3, 3–10 and 10–18 years). The second model considers weight and height as variables, is stratified by gender and applied to only the age range of 10–18 years. The *R* value for the correlation of the variables with energy expenditure was not mentioned. A correlation between *R* = 0.97 and *R* = 0.77 was found for the equations, and the equation models with the highest reported *R* values corresponded to the equations for both males and females aged 0–3 years that take into account the weight variable; the model with the lowest reported *R* value was that for the equation for females aged 10–18 years that takes into account the variables of weight and height.

The authors reported significant variation in the BEE related to ethnic differences, with 10% variation in the Indian population.

### Schofield Equation

The BEE prediction equations designed by Schofield (10) in 1985 were derived from databases including pediatric and adult populations that included European (mainly Italian), American and Asian subjects from developed and underdeveloped countries. From a sample of 2,359 children, two different equation models were developed for the prediction of BEE: the first model takes into account the weight variable, and the second model uses weight and height. Both models were stratified by sex and further subdivided by age (0–3, 3–10 and 10–18 years). The *R* value for the correlation of the variables used in the models with energy expenditure was not reported. *R* values between 0.97 and 0.81 were reported for the equations; the models with the highest *R* values were those corresponding to the equations for men and women aged 0–3 years that take into account weight and height. On the other hand, the model with the lowest *R* value reported was the model for women aged 3–10 years that takes into account the variables of weight and height. A small part of the database included children with illness (without specifying the number of subjects or the diseases), which the author reports was not a significant proportion of the sample.

### Henry and Collaborators' Equation, 1999

Henry et al. (11) designed a number of adolescent BEE prediction equation models in 1999, taking into account pubertal status, based on a sample size of 195 adolescents (78 males and 117 females) aged 10–15 years in Oxford, UK. Skinfold measurements were taken from 5 areas (the biceps, triceps, subscapular, suprailiac and mid-calf areas), which were used to establish percentage body fat (%BF), fat-free mass (FFM) and fat mass (FM). In addition, measurements of mid-upper arm muscle circumference (MUAMC) and wrist breadth were obtained. Sexual development was assessed by physicians, who compared the images and descriptions described in Tanner's puberty classification (pubic hair and gonadal development in males and breast development and the onset of menarche in females). Energy expenditure was measured by IC.

The authors established several equation models. Model 1 was stratified by gender; for males, the authors designed 5 equations that included variables such as weight, FFM, height, FM and skinfold measurements in different combinations, while for females, they designed 3 equations that included variables such as weight, height, FFM, age and FM in different combinations. Model 2 was stratified by gender and pubertal status. For males, 2 equations that included the variables of weight, age, wrist width, MUAMC and the logarithm of the sum of the skinfold measurements were established. For females, 4 equations that included variables such as weight, menarche status, FM and age were established.

The equation that had the highest *R*^2^ value (0.69) was the equation for males in gonadal pubertal stage 3 (G3) compared to the following equations, which had *R*^2^ values of 0.52: model 1 for females that included the weight variable, model 2 for females in the pubertal stage (breast stage 1) and finally the model for females aged 10–15 years.

The authors concluded that the inclusion of menarche status in the regression equations improved the estimation of BEE in premenarchal females. However, in males, pubertal stage, as assessed by pubic hair and gonadal stage, did not contribute to a significant improvement in BEE estimation, except in 11-year-old boys. That is, the inclusion of pubertal stage provided only minor improvements.

### IOM Equation, 2002

In 2002, the IOM (12) developed a BEE equation for children and adolescents with normal weight from a database of 525 children and adolescents, including 167 males (73 were of Caucasian, 13 were of African American, 4 were of Hispanic and 62 were of American Indian ethnicity) and 358 females (197 were of Caucasian, 58 were of African American, 20 were of Hispanic, 10 were of Asian and 60 were of American Indian ethnicity), aged 3–18 years, who were healthy and had energy expenditure measurements taken using the doubly labeled water technique. In addition, 20, 10 and 60 children and adolescents of Hispanic, Asian, and American Indian ethnicity who were aged 3–18, healthy and underwent energy expenditure measurements using the doubly labeled water technique were evaluated to establish two equations, which were stratified by gender. Within the equations, the variables of age, height and weight were considered. The *R* value for the correlation of the variables with energy expenditure was not reported. The equation for males had the highest *R*^2^ value, at 0.89, compared to 0.75 for the equation for women. All data were collected in the USA.

### Henry and Collaborators' Equation, 2005

From a compilation of 116 investigations, Henry (4) created a database of European, American and Asian populations of different age ranges in 2005. The authors evaluated a population of 10,552 persons (5,794 males and 4,702 females), including 4,018 subjects from the tropics, and excluded all Italian subjects. The number of children and adolescents included in the study was not specified.

The authors designed different BEE prediction equations models, known as the Oxford equations. Specifically, for the pediatric population, 2 equation models were derived, the first taking into account the weight variable and the second taking into account the weight and height variables. Both models were stratified by gender and subdivided by age (0–3, 3–10, and 10–18 years). The equations had *R*^2^ values between 0.964 and 0.752, corresponding to the weight and height equation for women aged 0–3 years and the weight equation for women aged 10–18 years, respectively.

### Lawrence and Collaborators' Equation

Lawrence et al. (13) designed several REE prediction equation models, known as Kim's equations, in 2009. They studied a sample size of 92 apparently healthy preschool children and third and fifth graders (38 boys and 54 girls) aged 4–11 years from a rural area of South Korea. None of the girls had begun menstruation. Body fat (BF) was assessed by bioelectrical impedance analysis (BIA), muscle mass was calculated using the Heymsfield formula (15), and BSA was calculated using the Dubois formula (16). Energy expenditure was measured by IC.

Four REE prediction equations were designed with different combinations of variables. Equation 1 takes into account percent ideal body weight (PIBW), BSA and BF; the second equation takes into account only BSA; the third equation takes into account age and weight; and the fourth equation takes into account only FFM. The *R* value for the correlation of each variable with energy expenditure was not documented. The equations had *R*^2^ values between 0.611 and 0.556, with the highest correlation for equation 1 and the lowest correlation for the model that takes into account only FFM.

### Kaneko and Collaborators' Equation

Kaneko et al. (14) established 2 of REE equation models in 2013; these models were designed from a sample of 221 Japanese children and adolescents (113 males and 108 females) aged 6–17 years who were apparently healthy and free of any condition affecting energy expenditure, such as abnormal thyroid gland function. BF was determined by two methods: BIA and skinfold measurements. FFM was calculated by subtracting BF from weight; however, this variable was not considered in the final model. Energy expenditure was measured by IC.

Two equations were obtained (one for each gender) that take into account the variables of weight, height and age. The equation designed for males had the highest *R*^2^ value, at 0.861, compared to 0.628 for the equation for women.

### Prediction Equations in Pediatric Populations With Overweight and Obesity

Seventeen articles were found with predictive equations with reference to pediatric populations with overweight and obesity. The predictive equations and their characteristics are presented in Table 3.

**Table 3:** Equations for the prediction of energy expenditure in the pediatric population with overweight or obesity.

**References**	***N***	**Clinical condition**	**Age range**	**(R^2^) Predictive equation**	**Calorimeter**	**Notes – Country where it was developed and/or population – Coding of variables of the equation – Body composition assessment method**
McDuffie et al. (17)	191 B 311 G	Normal weight and overweight	6–11 years	**Weight and height** **Boys (R^2^ = 0.72)** • REE (Kcal/d) = (0.037 *× W*) – (4.67 ×1/*H*^2^) – (0.159 × *R*) – 6.792. *Adjustment if predicted REE is ≤ 6.0 MJ, then REE = −0.217; else + 0.277* **Girls (R^2^ = 0.69)** • REE (Kcal/d) = (0.046 *× W*) – (4.492 ×1/*H*^2^) – (0.151 × *R*) + 5.841. *Adjustment if predicted REE is ≤ 5.0 MJ, then REE = −0.457; else + 0.244* **Body composition** **Boys (R^2^ = 0.75)** • REE (Kcal/d) = (0.078 × FFM) + (0.026 × FM) - (2.646 ×1/H^2^) – (0.244 × R) + 4.8. *Adjustment if predicted REE is ≤ 6.0 MJ, then REE = −0.255; else + 0.251* **Girls (R^2^ = 0.71)** • REE (Kcal/d) = (0.101 × FFM) + (0.025 × FM) + (0.293 × *H*^2^) – (0.185 × R) + 1.643. *Adjustment if predicted REE is ≤ 5.0 MJ, then* • •*REE =-0.355; else + 0.251*	SensorMedics 2900 or Deltatrac; SensorMedics Corp, Yorba Linda, CA	• Data from the National Institutes of Health Washington, DC; Philadelphia, PA; Pittsburgh, PA; and Baton Rouge, LA • R = Race (Black = 1/White = 0) • Body composition determined by DEXA
Dietz et al. (18)	No obesity 14 B 12 G Obesity 15 B 13 G 25 G	Normal weight and obesity	10–18 years	Boys (R^2^ = 0.79)* • BEE (kcal/d) = (16.6 *× W*) + (77 *× H*) + 572 Girls (R^2^ = 0.59)* • BEE (kcal/d) = (7.4 *× W*) + (482 *× H*) + 217 Girls (R^2^ = 0.84)* • BEE = 25.438 + (34.913 × FFM)	Not reported	•Boston, MA •FAO/WHO equation for weight and height for ages 10–18 years •Body composition measurement method was not documented
Tounian et al. (19)	No obesity 8 Obesity 19	Normal weight and obesity	11–17 years	**Control group (No obesity)** Girls (R^2^ = 0.60)* • REE (Kcal/d) = (23.2 × FFM) + 726 **Obesity** Girls (R^2^ = 0.50)* • REE (Kcal/d) = (18.1 × FFM) + 872.2 • REE (Kcal/d) = (24.7 × FFM) – (8.92 × BF) + 841	MMC Horizon-Beckman gas analyzer (SensorMedics Corp., Anaheim, CA)	• Paris, France • Body composition was calculated using the formula of Durnin and Rahaman
Maffeis et al. (20)	No obesity 97 Obesity 33	Normal weight and obesity	6–10 years	**Boys (R^2^ = 0.58)** • **6–10 years** → REE (kJ/d) = 1,287 + (28.6 *× W*) + (23.6 *× H*) – (69.1 *× A*) **Girls (R^2^ = 0.69)** • **6–10 years** → REE (kJ/d) = 1,552 + (35.8 *× W*) + (15.6 *× H*) – (36.3 *× A*)	Deltatrac calorimeter; Instrumentarium Oy, Datex Division, Helsinki, Finland	• Italy • Body composition was calculated using the Lohman formula
Molnár et al. (21)	No obesity 116 B 119 G Obesity 77 B 59 G	Normal weight and obesity	10–16 years	**Boys (R^2^ = 0.884)** • REE (kJ/d) = (50.0 *× W*) + (25.3 *× H*) – (50.3 *× A*) + 26.9 **Girls (R^2^ = 0.824)** • REE (kJ/d) = (51.2 *× W*) + (24.5 *× H*) – (207.5 *× A*) + 1,629.8 **Boys and Girls (R^2^ = 0.859)** • REE (kJ/d) = (50.2 *× W*) + (26.9 *× H*) – (144.5 *× A*) – (550 × *G*) + 594. 3	Deltatrac indirect calorimeter (Datex, Instrumentarium OY, Helsinki, Finland)	• Hungary • Gender: 0 boys and 1 girls • Body composition was determined by skinfold measurements according to Parizkova and Roth
Müller et al. (22)	243	Normal weight and obesity	5–17 years	**R^2^ = 0.72)** • **REE (MJ/d)** = (0.02606 *× W*) + (0.04129 *× H*) + (0.311 × G) – (0.08369 *× A*) – 0.808 **R**^**2**^ **= 0.72)** • **REE (MJ/d) =** (0.07885 × FFM) + (0.02132 × FM) + (0.327 × G) + 2.694	Deltatrac, TM MBM-100; Hoyer, Bremen, Germany	• Germany • Gender: 1 boys and 0 girls • Body composition was determined by BIA
Uemura et al. (23)	76	Normal weight and obesity	12–13 years	**Total equation (R^2^ = 0.65)** • REE (Kcal/d) = (40.2 × G) + (11.2 *× W*) + (9.6 *× H*) + (10.3 × FFM) – 767 **Equation with obesity (R^2^ = 0.55)** • REE (Kcal/d) = (23.7 *× W*) + (11.3 *× H*) – (10.7 × FM) – 1,162.3 **Equation with weight normal (R^2^ = 0.48)** • REE (Kcal/d) = (40.4 × FFM) + 146.4	Douglas 1911; Yamauchi and Ohtsuka 200	• Indonesia • Gender: 1 boys and 0 girls • Body composition was determined by BIA
IOM (12)	127 B 192 G	Overweight and obesity	3–18 years	**Boys (R^2^ = 0.88)** • BEE (kcal/d) = 419.9 – (33.5 *× A*) + (418.9 *× H*) + (16.7 *× W*) **Girls (R^2^ = 0.76)** • BEE (kcal/d) = 515.8 – (26.8 *× A*) + (347 *× H*) + (12.4 *× W*)	Doubly labeled water technique	• Caucasian, African American, Hispanic, and American Indian populations • Height in meters (m)
Tverskaya et al. (24)	50 B 60 G	Obesity	6–10 years	**(R^2^ = 0.84)** • BEE (Kcal/d) = 775 + (28.4 × FFM)–(37 *× A*) + (3.3 × FM) + (82 × G)	Deltatrac (Model MBM-100, SensorMedics Corp., Yorba Linda, CA)	• Brooklyn, New York • Gender: 1 boys and 0 girls • Body composition was determined by BIA
Derumeaux-Burel et al. (25)	191 B 280 G	Obesity	3–18 years	**Boys (R^2^ = 0.79)** • REE (Kcal/d) = (0.1096 × FFM) + 2.8862 **Girls (R^2^ = 0.76)** • REE (Kcal/d) = (0.1371 × FFM) – (0.1644 *× A*) + 3.3647	Deltatrac II apparatus (Datex Engström, Helsinki)	• France • Body composition was determined by BIA
Schmelzle et al. (26)	49 B 33 G	Obesity	4–15 years	Equation 1 (group 1 both) (R^2^ = 0.64)* • **4–10 years →** REE (kcal/d) = (38.8 × FFM_DEXA)_ + 505 Equation 2 (group 2 Boys) (R^2^ = 0.65)* • **11–15 years →** REE (kcal/d) = (27.2 × FFM_DEXA_) + 766**)** Equation 3 (group 3 Girls) (R^2^ = 0.65)* • **11–15 years →** REE (kcal/d) = (12.1 *× W*) + 689 Equation 4 (group 1 both) (R^2^ = 0.59)* • **4–10 years →** REE (kcal/d) = (15.0 *× W*) + (5.3 *× H*)−3 Equation 5 (group 2 Boys) (R^2^ = 0.57)* • **11–15 years →** REE (kcal/d) = (6.6 *× W*) + (13.1 *× H*)−794 Equation 6 (group 3 Girls) (R^2^ = 0.65)* • **11–15 years →** REE (kcal/d) = (11.9 *× W*) + (0.84 *× H*) + 579	Deltatrac I1 metabolic monitor (Datex, Finland)	• Germany • Body composition was determined by DEXA
Lazzer et al. (27)	242 B 332 G	Obesity	7–18 years	**(R^2^ = 0.66)** • REE (kJ/d) = (*G* ×892.68) – (*A* ×115.93) + (*W* ×54.96) + (*H* ×1,816.23) + 1,484.50 **(R^2^ = 0.66)** • REE (kJ/d) = (G ×909.12) – (A ×107.48) + (FFM ×68.39 + (FM ×55.19) + 3,631.23)	*V*_max_ 29; SensorMedics, Yorba Linda, CA, USA	• Italy, Caucasian population • Height in meters (m) • Gender: 1 boys and 0 girls • Body composition was determined by the de Lazzer equation and validated by DEXA
Chan et al. (28)	71 B 29 G	Obesity	7–18 years	**(R^2^** = **0.7**) REE (kcal/d) = (17.4 × logFFM) + (11.4 × ConI) – (2.4 × CenI) – 31.3	Deltatrac II MBM-200; Instrumentarium Corp, Helsinki, Finland	• China • ConI (conicity index) = waist circumference (m)/0.109 √ [P/T (in meters)] • CenI (centrality index = subscapular/triceps skinfold measurement) • Body composition by DEXA
Lazzer et al. (29)	1,412	Obesity	7–18 years	**Equation 1 (Adjusted** *R*^2^ **= 0.59)** • BEE (kcal/d) = (12 *× W*) – (14 *× A*) + (241 × *G*) + 909 **Equation 2 (Adjusted** *R*^2^ **= 0.59)** • BEE (kcal/d) = (24 × FFM) – (7 *× A*) + (179 × *G*) + 870	*V*_max_ 29; SensorMedics, Yorba Linda, CA	• Italy • Gender: 1 boys and 0 girls • Body composition was determined by BIA; FFM was estimated using the Lazzer et al. prediction equation
Lazzer et al. (30)	682 B 1014 G	Obesity	7–18 years	**(R^2^ = 0.69)** • BEE (MJ/d) = (*W* ×0.044) + (*H* ×2.836) – (pubertal stage ×0.148) + (*G* ×0.781) – 0.551 **(Adjusted** *R*^2^ **= 0.70)** • BEE (MJ/d) = (FFM ×0.082) + (FM ×0.037) - (pubertal stage ×0.125) + (*G* ×0.706) + 2.528	Vmax29, SensorMedics, Yorba Linda, CA, USA	• Caucasian population, Italy • Height in meters (m) • Pubertal stage: (1 = prepubertal to 5 = fully mature) • Gender: 1 boys and 0 girls • Body composition was determined by BIA; FFM was estimated using the prediction equation of Lazzer et al.
Acar-Tek et al. (31)	57 B 46 G	Obesity	7–17 years	(Adjsuted **R^2^ = 0.419)** • REE (Kcal/d) = 451.722 + (23.202 × FFM)	COSMED, FitMatePro, Rome, Italy	• Ankara, Turkey • Body composition was determined by BIA
Zhang et al. (32)	148	Obesity	7–13 years	**(R^2^ = 0.401)** • REE (Kcal/d) = 54.41 – (1.36 *× A*) – (2.25 × BMISDS) – (0.16 × FFM)	Not reported	• Chinese population • BMISDS = body mass index standard deviation score • Body composition was determined by BIA
Chu et al. (33)	26	Obesity	Adolescents	**(R^2^ = 0.730)** • REE (Kcal/d) = (10.733 × FM) + (12.727 × FFM) + 595.071	(*V*_max_ Encore V29C; SensorMedics Corp., Yorba Linda, CA)	• Toronto, Ontario • Reactance and reactivity were determined by BIA; body composition was determined by the equations of Gray et al.

*BEE, basal energy expenditure; REE, resting energy expenditure; B, boys; G, girls; W, weight (kg); H, height (cm); A, age (years); FFM, fat free mass (kg); FM, fat mass (kg); BF, body fat (kg); G, gender (sex), as reported in the original equation; DEXA: dual-energy X-ray absorptiometry; kcal, kilocalories; kJ, kilojoules; MJ, megajoules; BIA, bioelectrical impedance analysis*.

* *The equations with Pearson's R were converted by squaring its value to obtain the value of R^2^ to standardize the values*.

### McDuffie and Collaborators' Equation

McDuffie et al. (17) designed REE prediction equations for children with normal weight and overweight in 2004. A total of 502 children (191 males and 311 females) aged 6–11 years were studied. The data for 176 of these children (from Washington, DC) were derived from the National Institutes of Health. The data for 136 children from Philadelphia, 69 children from Pittsburgh and 121 children from Baton Rouge (LA) were derived from studies by four authors. Among the 502 children, 212 were black, and 290 were white. According to body mass index (BMI), 37.6% had normal weight (between the 5th and 84th percentiles), 10.9% had a risk of overweight (between the 85th and 95th percentiles) and 51.4% had overweight (>95th percentile) for age and gender. Disease was ruled out in the participants. Energy expenditure was measured by IC. Body composition (FM and FFM) was assessed by dual-energy X-ray absorptiometry (DEXA).

Two different models of prediction equations, stratified by gender, were developed. The first model takes into account the variables of weight, height and race, and the second model considers FFM, FM, height and race as variables. The *R* values for the correlations of the variables with energy expenditure was not mentioned. The equations had *R*^2^ values between 0.75 and 0.69; the highest *R*^2^ was for the equation for men that takes into account body composition, and the lowest *R*^2^ was the equation for women that considers only weight and height.

### Dietz and Collaborators' Equation

Dietz et al. (18) conducted research in Boston, MA, in 1990, in which they proposed that the FAO/WHO weight and height equation for the age range of 10–18 years is more accurate in the prediction of BEE in pediatric populations with obesity. They analyzed a sample of 54 adolescents with obesity (15 males and 13 females) and without obesity (14 males and 12 females). The degree of obesity was found to range from mild to severe; however, these values were not documented. The energy expenditure was measured by IC.

The authors also designed an equation for the prediction of BEE from a sample of 25 adolescent women with and without obesity in which FFM is the only variable; however, they did not document how FFM was assessed. The equation had an *R* value of 0.92.

### Tounian and Collaborators' Equation

In 1993 in Paris, Tounian et al. (19) established several REE prediction equations for girls with obesity. The authors studied 27 girls (19 with obesity and 8 controls). Among the group with obesity, the age range of the population studied was 11.8–17.1 years, and 13 of them were found to have a positive family history of obesity (defined as a BMI >90th percentile for age and gender in one or both parents). Obesity was determined by curves (not specified), and pubertal stage was determined according to Tanner staging. The diet of the group with obesity was controlled, while that of the control group was not. Skinfold measurements were taken at 4 sites (the biceps, triceps, subscapular and suprailiac areas), and FFM and BF were calculated using the Durnin and Rahaman formula for adolescent females (34). Energy expenditure was assessed by IC.

Three different equation models were developed. Two of the equations were derived from the group with obesity, with one taking into account the FFM and the other taking into account both FM and FFM. The third equation model was derived from the control group and takes only FFM into account. The latter equations had *R* values of 0.71 and *R* = 0.78, respectively.

### Maffeis and Collaborators' Equation

In 1992, Maffeis et al. (20) designed different REE prediction equations for a pediatric population with obesity in Italy. These authors evaluated a population of 130 healthy white prepubertal children aged 6–10 years, who were divided into 2 groups: a group of 97 children without obesity (body weight between 90 and 119% of the expected weight for height) and a group of 33 children with obesity (weight ≥20% of the expected weight for height). Those with diabetes mellitus or other metabolic and/or endocrine diseases were excluded. Prepubertal status was assessed according to Tanner staging. Skinfold measurements (tricipital and subscapular skinfold measurement in millimeters) were determined, and Lohman's formulas (35) were used to estimate relative BF. The FFM was calculated by subtracting FM from body weight. FM was obtained by multiplying %BF by body weight. Energy expenditure was assessed by IC.

Two final equation models were established, one for each gender. In both models, the variables considered to be correlated with energy expenditure were weight (*R* = 0.725 and *R* = 0.825), height (*R* = 0.684 and *R* = 0.722), and age (*R* = 0.480 and *R* = 0.577) for males and females, respectively. The equation with the highest *R*^2^ value, at 0.69, was the equation for females, compared to 0.58 for the equation for males.

### Molnár and Collaborators' Equation

Molnár et al. (21) established 2 prediction equation models of REE in 1994 in Hungary from a sample of 371 healthy adolescents, including 235 without obesity (116 males and 119 females) and 136 with obesity (77 males and 59 females); these adolescents were between 10 and 16 years of age and thus in the pubertal and postpubertal stages. Participants without obesity had a body weight <120% of the expected weight for height, while those with obesity exceeded the expected weight for height by 20% or more.

The authors assessed pubertal stage according to Tanner staging; however, this variable was not included in the models due to its low predictive power in the regression analysis. Energy expenditure was estimated by IC. Triceps, biceps, suprailiac, subscapular, suprascapular and calf skinfold measurements were taken. Relative BF was estimated from the five skinfold measurements according to Parizkova and Roth (36). FFM was calculated by subtracting BF mass (%BF × body weight) from body weight; however, none of these variables were considered in the final equation models.

The authors designed 2 equation models: model 1 was stratified by gender and model 2 included both sexes. The variables correlated with energy expenditure that were used in model 1 were weight (*R* = 0.928 and *R* = 0.862), height (*R* = 0.707 and *R* = 0.474) and age (*R* = 0.431 and *R* = 0.175) for males and females, respectively. Likewise, for model 2, weight (*R* = 0.881), height (*R* = 0.612), age (*R* = 0.294) and gender were used. The equation with the highest *R*^2^ value was the equation for males, with an *R*^2^ of 0.884, while the lowest *R*^2^ value was obtained for the equation for females, with an *R*^2^ of 0.824.

The equations of model 1 were validated in a second independent cohort of adolescents (80 males and 61 females) and were found to reliably estimate REE in adolescents with or without obesity who were aged 10–16 years; an individual error of the estimate of REE of <15% was reported for both equations.

### Müller and Collaborators' Equation

From a database covering 7 centers in Germany, Müller et al. (37) designed two equation models to predict REE in 2004. A population of 243 children and adolescents with mainly overweight and obesity aged 5–17 years was studied. Weight was classified as normal, overweight or obesity using German BMI percentiles (<10, >90, and >97, respectively). Body composition was assessed by BIA, and energy expenditure was assessed by IC.

Model 1 used the variables of weight, height, sex and age. Model 2 used the variables of FFM, MG and gender. The *R* values for the correlations between each of these variables and energy expenditure were not mentioned. Both equations had an *R*^2^ value of 0.72.

### Uemura and Collaborators' Equation

Uemura et al. (24) designed several REE prediction equations in 2011. These authors studied a population of 76 high school students (35 with normal weight and 41 with obesity) aged 12 and 13 years in Indonesia. Obesity was established according to BMI cutoff values developed by the International Obesity Task Force (25). High school students with a history of metabolic or endocrine diseases and taking regular medication were excluded. Energy expenditure was assessed by IC, and body composition was assessed by BIA.

Three predictive equations were established: the equation for the general population considered the variables of sex, weight, height and FFM; the equation for the population with obesity considered the variables of weight, height and FM; and the equation for the population with normal weight considered only FFM. The *R* value for the correlation of each variable with energy expenditure was not documented. The *R*^2^ value of the equation for the population with normal weight was 0.48, whereas that of the general-population equation was 0.65.

### IOM Equation, 2002

In 2002, the IOM (12) developed a BEE prediction equation for children and adolescents with overweight and obesity from a database of 319 children and adolescents with overweight and obesity, including 127 males (33 were of Caucasian, 20 were of African American, 2 were of Hispanic and 71 were of American Indian ethnicity) and 192 females (63 were of Caucasian, 48 were of African American, 6 were of Hispanic, 68 were of American Indian and 1 was of Asian ethnicity). Children and adolescents aged 3–18 years with BMIs >85th percentile were evaluated. Those who were receiving diet and exercise interventions were excluded. Energy expenditure was assessed using the doubly labeled water technique and stratified by gender, and two equations were established; within the equations, the variables of age, height and weight were considered. The *R* values for the correlations of these variables with energy expenditure were not reported. The equation for males had the highest *R*^2^ value, at 0.88, compared to 0.79 for the equation for women. All data were collected in the USA.

### Tverskaya and Collaborators' Equation

A BEE prediction equation for the pediatric population with obesity was designed by Tverskaya et al. (24) in Brooklyn, New York in 1998. The authors evaluated a total population of 110 pediatric patients (50 males and 60 females) aged 10–18 years with a BMI >28 kg/m^2^, of whom 81% were of Caucasian ethnicity, 11% were of Hispanic ethnicity, and 8% were of African American ethnicity. Of these, a sample of 100 subjects was used to design the equation, and the remaining 10 were used to validate the equation. Energy expenditure was assessed by IC, and body composition was assessed by BIA. The variables that correlated with energy expenditure and were taken into account in the equation were FFM and FM (*R*^2^ = 0.749 and *R*^2^ = 0.833, respectively), age (*R*^2^ = 0.811) and gender (*R*^2^ = 0.843). The final model of the equation had an *R*^2^ value of 0.84.

### Derumeaux-Burel and Collaborators' Equation

Derumeaux-Burel et al. (25) developed two REE prediction equations for children and adolescents with obesity. These equations were designed in 2004 on the basis of data from a sample of 471 French children and adolescents (191 males and 28.0 females) aged 3–18 years with a BMI *Z-*score ≥2 who visited the nutritionist for the first time; children and adolescents with any disease were excluded. Body composition [body mass (BM) and FFM] was assessed by BIA, and energy expenditure was assessed by IC.

Derumeaux-Burel et al. (25) established two equations. The first equation was for males and takes into account a single variable, FFM. The second equation is for females and takes into account FFM and age. The equation for males had an *R*^2^ value of 0.79, and the equation for females had an *R*^2^ value of 0.76. The equations were validated in an independent cohort of 211 children (62 males and 149 females).

### Schmelzle and Collaborators' Equation

In 2004, Schmelzle et al. (26) designed REE prediction equations for the pediatric population with obesity in Germany. A population of 82 subjects with obesity but who were otherwise healthy (49 males and 33 females) was studied. Obesity was diagnosed if the individual BMI exceeded the 95th percentile according to age- and sex-specific BMI tables. Individuals with underlying diseases, such as endocrinopathies or chromosomal abnormalities, were excluded. Energy expenditure was assessed by IC, and body composition (FM and FFM) was assessed by DEXA. Due to the sex-specific changes in body composition expected during puberty, the study group was divided into three groups: group 1, including boys and girls in the prepubertal stage (4–10 years); group 2, including boys aged 11–15 years; and group 3, including girls aged 11–15 years. Different REE equation models were established.

Schmelzle et al. (26) established 6 equations stratified by group and variable. The first 3 equations corresponded to groups 1, 2, and 3 and used only the FFM variable; these equations correlated with energy expenditure and had *R*^2^ values of 0.60, 0.63, and 0.55 in the whole population, male population and female population, respectively. The other 3 remaining equations used the weight and height variables; the *R*^2^ values of these variables for comparisons with energy expenditure were *R*^2^ = 0.58 and *R*^2^ = 0.50 for the whole population, *R*^2^ = 0.55 and *R*^2^ = 0.58 for the male population and *R*^2^ = 0.66 and *R*^2^ = 0.30 for the female population for weight and height, respectively. The equations had *R* values ranging from 0.76 to 0.81, with the highest correlation for the equation for men that takes FFM into account, followed by the equation for women that takes the weight variable into account and finally the equation for women that takes the weight and height variables into account.

### Lazzer and Collaborators' Equation, 2006

In 2006, Lazzer et al. (27) designed and validated two REE prediction equations for children and adolescents with obesity in Italy on the basis of data from a sample of 574 children and adolescents with Caucasian ethnicity and obesity (242 males and 332 females) aged 7–18 years. Those with a BMI above the 97th percentile for gender and age were included. Individuals who had previously participated in weight control programs, had metabolic and/or endocrine diseases, or were taking regular medication or any medication that influenced energy metabolism were excluded. FFM and FM were estimated using the prediction equations developed by Lazzer et al. (38) and BIA in a group of 143 adolescents with obesity (BMI *Z-*score: 3.2; % FM: 34.5) aged 12–17 years and validated by DEXA. Energy expenditure was determined by IC.

Two models including anthropometric and body composition parameters were constructed. The variables that correlated with energy expenditure were gender (*R*^2^ = 0.19), age (*R*^2^ = 0.05), weight (*R*^2^ = 0.74) and height (*R*^2^ = 0.32). The second model included gender, age, FFM (*R*^2^ = 0.66 for each of the variables) and FM (*R*^2^ = 0.41). Both equations had *R*^2^ values of 0.66.

The equations were internally and externally validated in an independent group of 53 adolescents with obesity.

### Chan and Collaborators' Equation

In 2009, Chan et al. (28) designed an REE prediction equation on the basis of data derived from a pediatric population with primary obesity; the authors evaluated a total population of 100 Chinese children (71 males and 29 females) aged 7–18 years. The authors included children with BMI above the 95th percentile according to local sex- and age-specific reference ranges with no evidence of underlying disease that could have caused secondary obesity detected during history taking or clinical examination. Children with obesity due to secondary causes were excluded.

The %BF was determined by skinfold measurements (from the biceps, triceps, subscapular and suprailiac areas). Assessments of obesity included the four skinfold area measurements and BMI. The measurement of central BF distribution included the waist-to-hip ratio and conicity index (ConI) (a function of waist circumference, weight and height), which was calculated as follows: waist circumference (m)/0.109 sq root [weight (kg)/height (m)]. The distribution of upper BM was demonstrated using the centrality index (CenI), which was calculated from the ratio of the subscapular skinfold measurement to the triceps skinfold measurement. The ideal BMI was considered to be in the 50th percentile according to age and sex references established by Cole, while the degree of obesity was presented as the percentage above the ideal BMI. Overweight and obesity were defined as age- and sex-specific BMIs corresponding to the cutoff points of 25 kg/m^2^ and 30 kg/m^2^, respectively, at 18 years of age. FFM was measured by DEXA, and energy expenditure was measured by IC.

A predictive equation was established with the variables FFM, ConI and CenI, and the *R* value for the correlation of each variable with energy expenditure was not documented; however, the *R*^2^ value for the equation was reported to be 0.7.

### Lazzer and Collaborators' Equation, 2010

In 2010, Lazzer et al. (29) designed BEE prediction equations for white children and adolescents with obesity in Italy, where a population of 1,412 children and adolescents aged 7-18 years was assessed. Children and adolescents with a BMI above the 97th percentile according to Italian reference values for gender and age were included. Those with metabolic and/or endocrine diseases or taking any medication influencing energy metabolism were excluded. Energy expenditure was assessed by IC, and body composition was assessed by BIA. FFM was estimated using the prediction equation of Lazzer et al. (39), FFM was estimated using the equations developed by Lazzer et al. (39), and FM was obtained by the subtraction of FFM from total weight and %BF was calculated as (FM/total weight) × 100.

Two models were constructed, the first taking the variables of weight, age and sex into account and the second taking the variables of FFM, age and sex into account. The *R* values of the correlations of the variables with energy expenditure were not reported. Both equations had an adjusted *R*^2^ of 0.59.

### Lazzer and Collaborators' Equation, 2014

Lazzer et al. (30) designed BEE prediction equations for children and adolescents with obesity in Italy in 2014 and included pubertal status. The authors evaluated a population of 1,696 Caucasian children and adolescents (682 males and 1,014 females) aged 7–18 years. Those with a BMI >97th percentile for gender and age were included. Those who had previously participated in weight control programs, had metabolic and/or endocrine diseases or were taking any medication that influenced energy metabolism were excluded.

Energy expenditure was assessed by IC. Pubertal stages (1 = prepubertal to 5 = fully mature) were assessed by palpation during a medical examination (pubic hair stages for both sexes, breast stages for girls and genitalia stages for boys). Body composition was assessed by BIA. FFM was estimated using the prediction equation of Lazzer et al. (39), and FM was obtained from the subtraction of FFM from total weight and %BF was calculated as (FM/total weight) × 100.

The first model takes the variables of weight (*R*^2^ = 0.56), height (*R*^2^ = 0.38), pubertal status (*R*^2^ = 0.21) and sex (*R*^2^ = 0.28) into account, and the second model takes the variables of FFM (*R*^2^ = 0.55), FM (*R*^2^ = 0.41), pubertal status at age and sex into account. Model 2 had the highest adjusted *R*^2^ value, at 0.70, compared to 0.69 for model 1.

### Acar-Tek and Collaborators' Equation

Acar-Tek et al. (31) designed REE prediction equations on the basis of data from a population of children and adolescents with obesity in Ankara, Turkey. A sample of 103 (57 males and 46 females) children and adolescents with obesity (BMI-for-age by *Z-*score ≥2 according to the WHO) aged 7–17 years was studied, excluding those with metabolic and thyroid dysfunction, respiratory diseases (asthma, influenza, or cold) and medication use. Body composition (BM, % fat and FFM) was assessed by BIA, while energy expenditure was measured by IC.

The equation proposed by Acar-Tek et al. (31) in 2017 considers only FFM, and an *R*^2^ of 0.470 was reported for the correlation with energy expenditure. The equation had an adjusted *R*^2^ value of 0.419.

An internal cross-validation analysis was performed. For adolescent girls, the difference between predicted and measured energy expenditure was −42 ± 266 kcal/d, and the equation had a prediction accuracy of 39.1% in this population; in the case of boys, the difference between predicted and measured energy expenditure was −32 ± 329 kcal/d, and the equation had a prediction accuracy of 43.9% in this population.

### Zhang and Collaborators' Equation

In 2018, Zhang et al. (32) developed an REE prediction equation for Chinese children with obesity. These authors evaluated a sample of 248 children, including 148 children with obesity aged 7–13 years. The group with obesity was established according to the body mass index standard deviation (BMISD) score, as established by the WHO. Participants who did not comply with fasting or became restless during the measurement or those who were taking medications such as thyroxine and prednisone that could potentially alter metabolic rate were excluded. Energy expenditure was assessed by IC. Body composition was measured with BIA. BMI values were transformed into a standard deviation score.

A prediction equation was established with the following variables, and their correlation with energy expenditure was reported: age (*R* = 0.41), BMISD (*R* = 0.19), and FFM (*R* = −0.53). The equation had an *R*^2^ value of 0.401.

### Chu and Collaborators' Equation

In 2019, Chu et al. (33) designed an REE prediction equation for adolescents with severe obesity in Toronto, Ontario, on the basis of preoperative data derived from a sample of 26 adolescents undergoing bariatric surgery. Energy expenditure was determined using IC. FM and FFM were estimated by BIA; they did not use the equation set in the device software but instead used resistance and reactance measurements for the equations developed by Gray et al. (40), as these equations more accurately predicted FM and FFM.

The equation developed takes into account the variables of FM and FFM, and although the *R* value for the correlation of each variable with energy expenditure was not documented, an *R*^2^ value of 0.730 was reported.

### Prediction Equations in the Pediatric Population With Specific Clinical Situations

Fourteen articles with predictive equations and reference to populations with various clinical situations and nutritional therapy, including sickle cell anemia, anorexia, muscular atrophy type 1, intensive care and surgical procedures, Stevens-Johnson syndrome (SJS), toxic epidermal necrolysis (TEN) and nutritional therapy, were found. The predictive equations and their characteristics are presented in Table 4.

**Table 4:** Equations for the prediction of energy expenditure in the pediatric population with specific clinical situations.

**References**	***N***	**Clinical condition**	**Age range**	**(R^2^) Predictive equation**	**Calorimeter**	**Notes – Country where it was developed and/or population – Coding of variables of the equation – Body composition assessment method**
Williams et al. (41)	6 B 14 G	Sickle cell anemia	5–17 years	**Harris-Benedict modified** **Boys** • REE (kcal/d) = [66.5 + (13.75 *× W*) + (5 *× H*) – (6.76 *× A*)] × (1.3278–0.0242 *× H*b) **Girls** • REE (kcal/d) = [655 + (9.56 *× W*) + (1.85 *× H*) – (4.68 *× A*)] × (13595–0.0242 *× H*b) **FAO/OMS modified** **Boys** • **3–10 years →** REE (kcal/d) = [(22.7 *× W*) + 495] × (1.3074–0.0309 *× H*b) • **10–18 years →** REE (kcal/d) = [(17.5 *× W*) + 651] × (1.3074–0.0309 *× H*b) **Girls** • **3–10 years →** REE (kcal/d) = [(22.5 *× W*) + 499] × (1.4775–0.0309 *× H*b) • **10–18 years →** REE (kcal/d) = [(12.2 *× W*) + 746] × (1.4775–0.0309 *× H*b)	CPX-MAX-D cardiopulmonary gas exchange system (Medical Graphics)	• Memphis, TN • Body composition was determined by BIA • Hb = hemoglobin (g/dl)
Buchowski et al. (42)	18 B 19 G	Sickle cell anemia	14–18 years	**Equation 1** **Both (R^2^ = 0.879)** • REE (kJ/d) = 3882 + (101 × FFM) – (439.8 × G=girls=) – (112.9 *× H*b) **Equation 2 (simple)** **Boys (R^2^ = 0.760)** • REE (kcal/d) = 1305 + (18.6 *× W*) – (55.7 *× H*b) **Girls (R^2^ = 0.855)** • REE (kcal/d) = 1100 + (13.3 *× W*) – (30.2 *× H*b)	Not reported	• Nashville, TN • Gender: 0 boys and 1 girls • Body composition was determined by hydrodensitometry. • Hb = hemoglobin (g/dl)
Scalfi et al. (43)	36	Anorexia nervosa	13–17 years	**Adolescents** (R^2^ = 0.484)* • BEE (kcal/d) = 313.4 + (100.8 *× W*) • BEE (kcal/d) = 92.8 *× W*	Canopy system: MMC Horizon, SensorMedics, Anaheim, USA	• Italy
Bertoli et al. (44)	49 B 73 G	Spinal muscular atrophy type 1	Under 10 years	**Spontaneous breathing** **(R^2^ = 0.630)** • REE (kcal/d) = (35 *× W*) + (75 × tx nusinersen) + 219 **(R^2^ = 0.630)** • REE (kcal/d) = (6 × SL) + (75 × tx nusinersen) + 10 **(R^2^ = 0.620)** • REE (kcal/d) = (24 × TL) + (97 × tx nusinersen) + 179 **Mechanical ventilation (R^2^ = 0.22)** • REE (kcal/d) = (14 × TL) +200 × tx nusinersen) + 190	VMAX Sensor Medics 29	• Caucasian population • Tx nusinersen: 1 = Yes • SL = supine length in centimeters (cm) • TL = tibia length in centimeters (cm)
Goran et al. (45)	56	Burn injury	4–14 years	**Resting energy expenditure** • REE (kcal/d) = 1.29 × PBEE **Energy required to ensure 95% of patients receive enough energy** • TEE (kcal/d) = [1.55 × PBEE + (2.39 × PBEE^0.75^)]	Beckman metabolic cart (Fullerton, CA)	• Texas • PBEE= prediction of basal energy expenditure (kcal)
Mayes et al. (46)	48	Burn injury	Under 3 years and 5–10 years	**Patients younger than 3 years of age** **(R^2^ = 0.71)** • REE = 108 + (68 × PW) + (3.9 × % burn) **(R^2^ = 0.68)** • REE = 179 + (66 × PW) + (3.2 × % third-degree burn) **Patients 5 to 10 years of age** **(R^2^ = 0.70)** • REE = 818 + (37.4 × PW) + (9.3 × % burn) **(R^2^ = 0.67)** • REE = 950 + (38.5 × PW) + (5.9 × third-degree burn)	Delta Trac, SensorMedics, Yorba Linda, CA	• Cincinnati, OH • Applicable for burns covering 10 to 50% of BSA • PW = preburn weight in kg • Percentage of BSA with 3rd degree burns (%)
White et al. (47)	58 B 42 G	Critical illness and ventilation	54 ± 53 months	**Equation 1 (R^2^ = 0.898)** • EE (kJ/d) = (20 *× A*) + (31 *× W*) + (151 *× W*AZ score) + (279 × Temp) + (122 × days UCI) – 9200 + constant **Equation 2 (simplified) (R^2^ = 0.867)** • EE (kJ/d)= (17 *× A*) + (48 *× W*) + (292 × Temp) – 9677	Deltatrac II (Datex-Engstrom, Helsinki, Finland)	• Brisbane, Australia • Age in months • WAZ score= Weight-for-age *Z-*score • ICU days = the number of days since admission to the ICU (if > 4, then multiplied by 4) • Temp = body temperature (°C) • **Constant=** + 0 (head injury); + 105 (postsurgical procedure);−512 (respiratory disease); + 98 (other);−227 (sepsis)
Meyer et al. (48)	175	Critical illness and ventilation	3–16 years	**Equation A (R^2^ = 0.833)** •** <3 years** → REE (kcal/d) = 309 + (48.4 *× W*) + (1.22x A) – (0.377 × *W*^2^) – 283.7 + (6.2 *× A*) + Dxcat – (Dxcatw *× W*) • **3–10 years** → REE (kcal/d) = 309 + (48.4 *× W*) + (1.22 *× A*) – (0.377 × *W*^2^) + 259 – (7.6 *× A*) + Dxcat – (Dxcatw *× W*) • **11–18 years** → REE (kcal/d) = 309 + (48.4 *× W*) + (1.22x A) – (0.30.377 × *W*^2^) + diagnosis coefficient* - (diagnosis coefficient *× W*) **Equation B (R^2^ = 0.839)** •** <3 years** → REE (kcal/d) = (87.5 *× W*) – 66 + Dxcat – (0.727 × *W*^2^) – (33 *× W*) • **3–10 years** → REE (kcal/d) = (87.5 *× W*) + 20 + Dxcat – (0.727 × *W*^2^) – (37.4 *× W*) • **11–18 years** → REE (kcal/d) = (87.5 *× W*) – 984 + Dxcat – (0.727 × *W*^2^) **Equation C (R^2^ = 0.829)** •** <3 years** → REE (kcal/d) = (88 *× W*) + 92 – (0.7 × *W*^2^) – (37 *× W*) • **3–10 years** → REE (kcal/d) = (88 *× W*) + 110 – (0.7 × *W*^2^) – (37 *× W*) • **11–18 years** → REE (kcal/d) = (88 *× W*) – 910 – (0.7 × *W*^2^)	Deltatrac II NMN-200 (Datex Ohmeda, Helsinki, Finland)	• London, UK **Equation A** **Dxcat =** **Diagnostic category** Multiorgan failure = 226 Respiratory failure = 79 Central nervous system = 33 Surgery = 0 **Dxcatw =** **Diagnosis category +** **weight in kg** Multiorgan failure = 18 Respiratory failure = 8 Central nervous system = 10 Surgery = 0 **Equation B** **Dxcat =** **Diagnostic category** Multiorgan failure = 143 Respiratory failure = 168 Central nervous system = 114 Cardiovascular and surgery = 142 Liver disease = 0
Mehta et al. (49)	72	Critical illness and ventilation	Under 18 years	REE (kcal/d) = 5.534 × VCO_2_ ×1,440	*V*_max__ Encore (Viasys Healthcare, Loma Linda, CA)	• Boston, MA • VCO_2_= Volume of carbon dioxide (L/min)
Jhang and Park (50)	32 B 38 G	Critical illness and mechanical ventilation	5 −17 years	**(R^2^ = 0.865)** • EE (kcal/d) = −321.264 + (72.152 *× W*) – (1.396 × *W*^2^) + (5.668 *× H*) + organ dysfunction*	CARESCAPE Monitor B650; GE Healthcare Finland Oy, Helsinki, Finland	• Korea • *hematologic= 76.699 • *neurologic = 87.984
Pierro et al. (51)	24 B 22 G	Gastrointestinal surgery	Under 6 months	(R^2^ = 0.84)* • REE (cal/min) = −74.436 + (34.661 *× W*) + (0.496 *× H*eart rate in beats/min) + (0.178 *× A*)	Taylor Servomex, Sussex, UK	• Liverpool, UK • Age in days • Conversion from cal/min to kcal/kg/d—the result of the equation is multiplied by 1.44 and divided by the weight in kg
Mayes et al. (52)	15	Stevens-Johnson syndrome and toxic epidermal necrolysis	9–12 years	**(R^2^ = 0.73)** • REE (kcal/d) = (24.6 × PW) + (wound size (%) ×4.1) + 940	DeltaTrac, SensorMedics, Yorba Linda, CA	• Cincinnati, OH • PW = Preinjury weight (kg) • Wound size (%) = percentage of the wound in relation to the total body surface area
Salas et al. (53)	37	Parenteral nutrition	2–7 years	(R^2^ = 0.974)* • REE (kcal/d) = (54.4 × FFM) + (0.095 × Creatinine/Weight) + 4.67	MMC Horizon (SensorMedics Metabolic Measurement Cart, Beckman Instruments, Inc., Anaheim, CA)	• Paris, France • Body composition measured by Brook's equation and Siri's equation based on skinfold measurements • Creatinine/weight in mmol/kg/d
Moukarzel et al. (54)	12 B 14 G	Parenteral nutrition	38–62 months	**Equation 1 (R^2^ = 0.98)** • REE (kcal/d) = (56.6 × FFM) + 97.9 **Equation 2 (R^2^ = 0.92)** • REE (kcal/d) = (45.6 *× W*) + 136 **Equation 3 (R^2^ = 0.985)** • REE (kcal/d) = (68.9 × FFM) + (3.3 × FM)	MMC Horizon metabolic cart (Beckman Instruments Inc., Anaheim, CA)	• Paris, France • Body composition measured by Brook's equation and Siri's equation based on skinfold measurements

BEE, basal energy expenditure; REE, resting energy expenditure; TEE, total energy expenditure; EE, energy expenditure, B, boys; G, girls; W, weight (kg); H, height (cm); A, age (years)*; FFM, fat-free mass (kg); FM, fat mass; G, gender (sex), as reported in the original equation; kcal, kilocalories; kJ, kilojoules; cal, calories.

* *To standardize the units, the equations with Pearson's R values were converted by squaring this coefficient to obtain the value of R^2^*.

### Williams and Collaborators' Equation

In 2002, Williams et al. (41) developed REE prediction equations in Memphis, Tennessee, by modifying the Harris-Benedict and FAO/WHO equations. The authors evaluated a sample of 20 children (6 males and 14 females) aged 5–17 years with sickle cell disease who had their energy expenditure assessed by IC and their BM and FFM determined by BIA.

Two models based on the Harris-Benedict equations, to which the hemoglobin (Hb) variable was incorporated, were reported. Moreover, the FAO/WHO equation models for the 3–10 and 10–18-year groups were modified to include the Hb variable. However, neither the *R* values nor the *R*^2^ values of the variables for the equation models were reported.

### Buchowski and Collaborators' Equation

Buchowski et al. (42) designed REE prediction equations for adolescent patients with sickle cell disease in Nashville, Tennessee, on the basis of data from a sample of 37 patients of African-American ethnicity (18 males and 19 females) with a confirmed diagnosis of sickle cell disease aged 14–18 years who were stable, i.e., no sickle cell crises during the study or for 28 days prior to the study and no metabolic, skeletal, hepatic or renal abnormalities. Energy expenditure was assessed by IC, and FM and FFM were determined by hydrodensitometry.

Two models were established: the first model takes into account FFM and Hb, and the second model is based on 2 equations stratified by sex and takes into account weight and Hb. The *R*^2^ value for FFM and weight in energy expenditure was documented, with *R*^2^ values of 0.805 and 0.702 for men and 0.757 and 0.825 for women, respectively. The *R* value for Hb was not documented. The highest *R*^2^ value, at 0.879, was reported for the equation for males.

### Scalfi and Collaborators' Equation

In 2001, Scalfi et al. (43) designed a BEE prediction equation for adolescents with anorexia nervosa in Italy. A sample of 34 adolescents aged 13–17 years who met the established criteria for the diagnosis of anorexia nervosa (Diagnostic and Statistical Manual of Mental Disorders, fourth edition (DSM IV) criteria) and amenorrhea for at least 6 months prior to testing were evaluated. These adolescents did not smoke, nor did they use any contraceptives or drugs that could affect energy expenditure, and they had not received any psychiatric or dietary treatment for at least 2 months prior to testing.

Energy expenditure was measured by IC and was predicted according to the formula of Schebendach et al. (55) (a correction of the Harris-Benedict equation for this condition). Weight and height measurements were performed.

Two prediction equations were established with weight as the single variable: the first equation had an *R* value of 0.696, while the *R* value for the second equation was not reported.

### Bertoli and Collaborators' Equation

In 2020, Bertoli et al. (44) designed different REE prediction equations in a population with spinal muscular atrophy type 1. With data derived from 5 reference centers in Italy, the authors evaluated a sample of 122 Caucasian children (49 males and 73 females) under 10 years of age with a clinical and genetic diagnosis of muscular atrophy type 1. Patients with a body weight >5 kg, with the absence of acute infection and who were not participating in experimental pharmacological protocols were included. Patients had received more than 4 loading doses of nusinersen. Patients with hemodynamic or respiratory instability or on ventilatory support with a fractional inspiratory oxygen fraction (FIO_2_) > 0.6 or positive end-expiratory pressure (PEEP) > 10 cm H_2_O were excluded.

The anthropometric variables assessed were weight, supine and tibial length and BMI. Clinical variables included the type of respiration (spontaneous or mechanical ventilation (non-invasive or invasive tracheostomy), the type of feeding (oral, nasogastric tube or gastrostomy) and nusinersen treatment (yes: treated (≥4 infusions) or no: untreated). Energy expenditure was assessed by IC.

Weight-, length-, and sex-specific BMI *Z-*scores were obtained using WHO growth standards. BMI-for-age values according to standard deviation cutoff points were classified as follows: below −1.644 (5th percentile), underweight; between −1.644 and +1.036, normal weight; between +1.036 and +1.644, overweight; and above +1.644, obesity.

The authors established 4 equation models, with nusinersen treatment as a common variable. The first 3 models were established for patients with spontaneous breathing and considers the variables of weight, supine length and tibia length. The fourth model was established for patients receiving mechanical ventilation and considers the tibia length variable. The *R* values for the correlations between the variables and energy expenditure were not reported.

The equations with the highest *R*^2^ value, at 0.63, were the models considering body weight, nusinersen treatment and both supine length and nusinersen treatment for patients with spontaneous breathing; the lowest *R*^2^ value, at 0.22, was reported for the equation for patients receiving mechanical ventilation.

### Goran and Collaborators' Equation

In 1991, Goran et al. (45) designed prediction equations for TEE and REE for patients with burns on the basis of a retrospective analysis of REE data obtained from 56 children aged 4–14 years in Texas. The variables considered for the prediction of REE were predicted basal energy expenditure (PBEE), BSA, age, weight, % of BSA burned and days after burn. BEE was predicted by the Harris and Benedict equations, BSA was calculated from height and weight, and % of BSA burned was calculated by observation on admission. Energy expenditure was measured by IC when continuous feeding was administered, and conditions such as fever, infection, antibiotics, analgesics, etc., were not controlled for at the time of measurement to reflect usual clinical conditions.

It was established that the PBEE variable was the best predictor for REE, with an *R*^2^ value of 0.76. Two equations were established, one to predict REE, which considers PBEE as the only variable, and another to predict TEE, which considers PBEE as the only variable. However, in the equation to predict TEE, another equation was added to determine PBEE to predict the energy required to ensure that 95% of patients received the energy needed to achieve energy balance. The first equation uses an activity factor of 1.2, which was derived from a previous study by the authors where the doubly labeled water technique was used for burn patients. Neither the *R* value nor the *R*^2^ value of the final models was mentioned.

### Mayes and Collaborators' Equation, 1996

In 1996, Mayes et al. (46) designed different REE prediction equations for children with burns in Cincinnati, OH. The authors evaluated a sample of 48 children who were divided by age into two groups (under 3 years and 5–10 years) and were randomized according to the % of BSA burned and the % of BSA with 3rd degree burns. Children who met the age criterion of each group, who were admitted within the first 10 days after burn injury, who received tube feeding within 24 h of admission and who had metabolic measurements determined on admission and weekly were included. Energy expenditure was measured by IC.

Four prediction equations were developed, stratified by age (under 3 and 5–10 years). An equation was developed for each age stratum mentioned above and considers the variables of preburn weight and % of BSA burned; another equation was developed for each age stratum, but this equation considers the variables of preburn weight and the % of BSA with 3rd degree burns. The *R* values for the correlations of the variables with energy expenditure were not mentioned. The equations had *R*^2^ values between 0.71 and 0.67, with the highest *R*^2^ value for the equation for patients younger than 3 years that considers the burn percentage variable and the lowest *R*^2^ value for the equation for patients aged 5–10 years that considered the % of BSA with 3rd degree burns. The equations are applicable for patients with burn injury covering 10–50% of their BSA.

### White and Collaborators' Equation

Prediction equations for delivered energy were designed by White et al. (47) for intensive care patients on the basis of data from a sample of 100 patients who were critically ill (58 males and 42 females), receiving mechanical ventilation and aged 54 months +/− 53 months in the pediatric intensive care unit (ICU) in Brisbane, Australia. Patients who had recent (<90 min) changes in ventilator variables or anesthetic gas administration or were receiving dialysis were excluded. The main reason for admission was classified according to clinical status: head injury, postsurgical procedure, respiratory disease, sepsis and others. Energy expenditure was measured by IC.

In 2000, White et al. (47) proposed two equation models. In the first equation, the variables that correlated with energy expenditure for which cumulative coefficients of determination were reported were age (*R*^2^ = 0.804), weight (*R*^2^ = 0.847), weight-for-age *Z-*score (WAZ score) (*R*^2^ = 0.867), temperature (*R*^2^ = 0.88), days in the ICU (i.e., the number of days since admission; if >4, multiplied by 4) (*R*^2^ = 0.902) and admission ratio (*R*^2^ = 891). The second equation, a simplified version of the first equation, takes into account only weight and temperature. The *R*^2^ value was 0.898 for equation 1 and 0.867 for the simplified equation.

Additionally, 16 males and 9 females (*n* = 25) were included in the validation dataset, and it was concluded that the equations are not suitable for children under 2 months of age.

### Meyer and Collaborators' Equation

Meyer et al. (48) designed different equation models to determine REE in children with critical illness admitted to ICUs in London, UK, from a database of three centers; of these, two specialized in cardiac disorders, infectious diseases, respiratory failure and neurodevelopmental disorders, while the third specialized in liver failure. Children with critical illness and receiving ventilation (term birth to 16 years) were included, while those receiving bolus enteral feeding; receiving renal replacement therapy; with endotracheal tube leakage of more than 10%; with an FiO_2_ >0.6; and ventilated with heliox, nitric oxide, or high-frequency oscillatory ventilation, were excluded. Age (<3, 3–10 and 11–18 years) and diagnosis on admission (multiorgan, respiratory, cardiac, central nervous system (CNS), gastrointestinal, surgical or hepatic failure) were categorized for analysis. The authors included a population of 175 children with IC energy expenditure assessment results, mainly with respiratory and CNS diagnoses, excluding gastrointestinal disease due to sample size.

In 2012, Meyer et al. (48) established 3 equation models (A, B, and C); the variables considered for the equations were weight, age and diagnosis. The *R* value for the correlation of each of them with energy expenditure was not specified. The *R*^2^ values ranged from 0.829 (simplified model C) to 0.833 (model B).

### Mehta and Collaborators' Equation

In 2015, Mehta et al. (49) designed an REE prediction equation based on a modification of the Weir equation considering a cohort from a multicenter study in Boston, MA. The authors evaluated a sample of 72 pediatric patients under 18 years of age receiving mechanical ventilation. The authors included patients in the ICUs of two centers who underwent gas exchange measurement and respiratory quotient (RQ) acquisition by IC; patients had been receiving enteral or parenteral nutrition, which was discontinued at the time of measurement. Patients with IC tests with an RQ outside the physiological range (>1.3 or <0.67) were excluded. Steady state was defined as a period of at least 5 min with <10% fluctuation in oxygen consumption (VO_2_) and carbon dioxide elimination (VCO_2_) and <5% fluctuation in the RQ.

The modified Weir equation was used to generate the simplified equation. The mean RQ was determined from the derivation data set. VO_2_ in the modified Weir equation was then replaced with VCO_2_/RQ to derive the simplified equation (VCO_2_-REE), which included only the VCO_2_ value. Validation of the equation was performed in a sample of 94 patients. Neither the *R* value nor the *R*^2^ value of the equation model was reported.

### Jhang and Collaborators' Equation

The prediction equation for energy expenditure constructed by Jhang and Park (50) in 2020 for intensive care patients was based on a sample of 70 Korean children (32 males and 38 females) who were critically ill, receiving mechanical ventilation and aged 5 months to 17 years. Those with an FiO_2_ > 60% while under mechanical ventilation, a respiratory rate >35/min, a tidal volume <35 ml, an air leak, a chest tube, continuous renal replacement therapy or extracorporeal membrane oxygenation, or severe fluid leakage by pleural or peritoneal drainage; those who were admitted to the ICU for <1 day; those with incomplete IC measurement; or those younger than 1 month or older than 18 years were excluded. Energy expenditure was measured by IC.

An energy expenditure equation was established using the variables of weight, height and organ dysfunction (hematological and neurological). The *R* values for the correlations of each variable with energy expenditure were not documented, but an *R*^2^ value of 0.865 was documented for the final model of the equation.

Twenty-five patients (14 males and 11 females) from a cohort were included for the validation of the equation, which showed less bias (15.51 kcal/day) and better percent agreement with the IC measurement (102.30% ± 28.10%) than other equations in use in pediatrics, such as the Schofield (10), Oxford (4), and FAO/WHO equations (9).

### Pierro and Collaborators' Equation

Pierro et al. (51) developed an REE prediction equation for stable infants undergoing surgery in Liverpool, UK, on the basis of data from a sample of 46 (24 males and 22 females) infants under 6 months of age who required a surgical procedure for gastrointestinal abnormalities and were receiving total parenteral (28 infants) or mixed parenteral and enteral (18 infants) feeding. Those with sepsis, congenital metabolic disorders, congenital heart defects and those requiring ventilatory support were excluded. Additional analyses were performed in 9 infants to validate the predictive value of the equation.

The variables that correlated with energy expenditure and are taken into account in the equation are weight (*R* = 0.87), heart rate (*R* = 0.60) and age (*R* = 0.49). The equation proposed by Pierro et al. (51) in 1994 had an *R* value for the correlation with energy expenditure of 0.92. The result is expressed in calories per minute. To convert to kcal/kg/d, the result of the equation is multiplied by 1.44 and divided by weight.

The equation can be applied to predict basal energy requirements in stable infants undergoing surgery from birth to 5 months of age.

### Mayes and Collaborators' Equation, 2008

An REE prediction equation was designed by Mayes et al. (52) in 2008 for patients with SJS and TEN in Cincinnati, OH. The data of 15 patients aged 9–12 years with a diagnosis of SJS and TEN who met the inclusion criterion of admission within 10 days of peel initiation were retrospectively evaluated. Energy expenditure was measured by IC.

A prediction equation was established with the variables of preinjury weight and wound size as a % of total BSA. The *R* values for the correlations of each variable with energy expenditure were not documented, but an *R*^2^ value of 0.73 was documented for the final equation model.

### Salas and Collaborators' Equation

In 1990 in Paris, France, Salas et al. (53) designed an REE prediction equation for children receiving total parenteral nutrition (TPN). The authors studied a total sample of 37 children ranging in age from 2 to 7 years, and the population was divided into two groups, A (*n* = 14) and B (*n* = 23), who were defined according to their weight-to-height ratio (weight/height) as <90 and >90%, respectively (ideal = 100%). The weight-for-height index represents weight as the percentage of children with normal weight and the same height and is an accurate index of nutritional status. No child in this study had a weight-for-height index >110%.

Those with the following pathologies were included: intractable diarrhea of childhood, Crohn's disease, liver disease, short bowel syndrome, and others (unspecified). Those with infection or renal failure were excluded. The authors performed skinfold measurements in 3 areas (the triceps, subscapularis biceps and suprailiac areas). They used Brook's equation (56) to determine body density from the sum of skinfold measurements. The %BF was derived from body density by using Siri's (57) equations (% BF= (4.95/density)−4.5), where BF is the product of weight and % BM. BFM is the difference between body weight and BM. Twenty-four-hour urine collections were taken on the same day as the energy expenditure measurement. Creatinine in 24-h urine was measured using an analytical procedure based on the Jaffe reaction (58). Twenty-four-hour urinary nitrogen was measured by the Kjeldahl method (59).

Energy expenditure was measured by IC, and continuous REE measurements were taken for 5.9 ± 2.4 h in group A and 5.5 ± 2.2 h in group B. During the recorded days, the REE of children receiving continuous TPN was measured for three periods of 3 h each. For patients receiving cyclic TPN, recording was performed for 3–4 h at 6 h after TPN was discontinued.

Correlations with REE were reported for the variables FFM (*R* = 0.984) and creatinine/weight (*R* = 0.394); for the final equation model, an *R* value of 0.987 was documented.

### Moukarzel and Collaborators' Equation

Prediction equations for REE in patients receiving TPN were designed by Moukarzel et al. (54) in 2003. The authors evaluated a sample of 26 children (12 males and 14 females) from a department of pediatrics in Paris, France. The patients ranged in age from 38 to 62 months and were receiving TPN (at least 3 months) due to diarrhea (*n* = 20) or short bowel syndrome (*n* = 6). Patients were stable and had good nutritional status (weight-for-height index). Skinfold measurements were taken in 4 areas (the biceps, triceps, subscapular and suprailiac areas).

The authors performed skinfold measurements in 4 areas (the triceps, subscapular biceps and suprailiac areas). The authors used Brook's equation (57) to determine body density from the sum of skinfold measurements. %BF was derived from body density using Siri's equation (58). FFM was the difference between weight and BM. Energy expenditure was assessed by IC.

The authors established 3 equation models: the first model uses the variable of FFM, the second model uses the variable of weight, and the third model uses the variables of FFM and FM. The *R* values for the correlations between the variables and energy expenditure were not reported. The equation that had the highest *R*^2^ value was the one that used the variables of FFM and FM, with *R*^2^ = 0.985.

## Discussion

We present a review of 39 studies in which equations were designed to predict energy expenditure in pediatric patients. Here, the *R*^2^ value was evaluated to determine the best model. It was shown that in healthy children who were described as having no significant medical problems, the FAO/WHO and Schofield equations had the highest *R*^2^ values and were simple equations that could be applied to a wide age range (3–18 years). The FAO/WHO equation was derived from a study population including pediatric patients in developed and underdeveloped countries, including Latin American countries, unlike the Schofield equation, which was derived mostly from the Italian population (11, 12).

In adolescent children with obesity, the equation of Molnár et al. (21) had the highest *R*^2^ value and is recommended for its simplicity, as it requires only weight, height and age data. Likewise, the equation of Dietz et al. (18) is recommended for older-age pediatric patients (10–18 years), as it has easily obtainable variables (weight and height) and good correlation with energy expenditure. Other equations, such as that of Tverskaya et al. (24), had high *R*^2^ values but used variables such as FFM and FM, which require specialized equipment that is difficult to obtain.

On the other hand, in patients with specific clinical conditions, the simplified equation of Meyer et al. (48) was demonstrated to be useful in patients receiving mechanical ventilation, as it had a good *R*^2^ for patients with critical illness aged 3–18 years and was simple to use since it requires only weight. Other authors proposed two equations for patients with sickle cell disease. Buchowski et al. (2) proposed two models: one is a simple equation with a good *R*^2^ value, and the other requires FFM data. On the other hand, in children post surgery, a good *R*^2^ value was observed for the Pierro et al. (51) equation, which utilizes variables that are easy to obtain; however, the main limitation of this equation was the age range (5 months). There were two equations proposed for patients receiving parenteral nutrition support, those of Salas et al. (53) and Moukarzel et al. (54); both had high *R*^2^ values.

Of the 91 models, body weight was used in 53, height was used in 26, age was used in 26 and sex was used in 13. These variables are considered easy to obtain in a doctor's office or any hospital. Other parameters that are considered in the equations are BSA, wrist circumference, MUAMC, menarche status, skin fat, body temperature, PIBW, WAZ score, days in the ICU, diagnostic category or coefficient, Hb and heart rate, all of which can be obtained without the use of any expensive instruments. Regarding body composition, FFM was used in 30 models, and FM was used in 11; however, these measurements were performed by skinfold measurements, BIA or DEXA, and the latter are not very accessible and may require trained personnel.

It is important to note that the Harris-Benedict equation, which was developed for the adult population, was included in the present review because it is one of the earliest equations generated (1918) and is the most widely used, even in the pediatric field. Two systematic reviews documented that the Harris-Benedict equation was included for the assessment of accuracy and precision in pediatric populations with obesity and critical illness (60, 61). These reviews have even been used as a basis for designing new equations modified from the Harris-Benedict equation (7).

It is important to consider possible bias induced by variables whose estimation or measurement, in clinical practice, may be different from those proposed and consider in the equation methodology, i.e., body composition (FM and/or FFM) evaluated by DEXA, as in the equation of Schmelze et al. (26), and clinical skinfold measurements, for their estimation and subsequent application in the equation. Future studies could consider the external validity of this adaptation.

An important process in the generation of energy expenditure prediction equations is the external validation of the equation, which must be performed in a population that is different from the one in which the equation was derived but shares the characteristics of the target population; of the studies in this review, only 9 mentioned the development and validation of the formula (21, 24, 27, 31, 47, 49–51); however, equations such as the FAO/WHO, Schofield, Harris and Benedict and Henry equations have been validated post publication and in various pediatric populations (2). In this systematic review, no equations that focused on populations with cancer, renal disease or liver disease were identified; this issue is important because some of these equations have been documented to have significant bias when used in the cancer population, for example (62).

This is the first known systematic review to date that reviews most of the equations designed for healthy pediatric populations and those with disease and focuses mainly on the development of each equation, with emphasis on its value in determining the final model. Thus, a strength of the study is that it summarizes the design process of each equation, which allows clinicians to broaden the criteria for choosing which equation to use in the clinical setting. However, the limitation of this study was that some published and commonly used equations had only *R* values and did not determine the *R*^2^; therefore, the conversion of *R* values to *R*^2^ values was performed to jointly evaluate the models. Another limitation of this study is that correlation coefficients were used to compare measured and predicted values of energy expenditure. This analysis merely assesses the relationship between measured and predicted values and does not assess the closeness of the predicted values to measured values.

## Conclusion

A wide variety of equations for the prediction of REE and BEE in the pediatric population have been published, and these equations are very heterogeneous in terms of the population in which they were established as well as the variables used in the prediction models. Although variables such as body weight, height, age, and gender were used in most equations, variables that are less accessible, such as body composition and Hb, were used in other equations. In clinical practice, precision is more important than accuracy of the equations, especially when measuring longitudinally; however, the magnitude of the accuracy of the equations should not be neglected. Important information included in this review of REE and BEE predictive equations for the pediatric population is the compendium of most of the equations; the population in which each equation was constructed (characteristics) can be observed, as well as the variables used in each equation and the values of adjustment considering the reference standard. Therefore, a critical evaluation of which equation should be used depending on the type of patient in clinical practice can be performed, allowing improvements in the estimates of energy requirements.

## Data Availability Statement

The original contributions presented in the study are included in the article/Supplementary Material, further inquiries can be directed to the corresponding author/s.

## Author Contributions

MG-C and IM-V designed the research. JF-S, AA-N, LG-S, OP-G, MS-R, AS-Z, IM-V, and MG-C performed the research and drafted the manuscript. JF-S, AA-N, LG-S, IM-V, and MG-C analyzed the data. All authors contributed to the article and approved the submitted version.

## Funding

This work was supported by Consejo Nacional de Ciencia y Tecnología (México) Grant: CONACYT-FRONTERAS 2019 (501225/2020). Monetary sponsorship was received from Hospital Regional de Alta Especialidad de la Península de Yucatán, Mérida, Yucatán, Departamento de Fisiología de la Nutrición, Instituto Nacional de Nutrición y Ciencias Médicas y Nutrición Salvador Zubirán, Ciudad de México, México, and Departamento de Metodología de la Investigación, Instituto Nacional de Pediatría, Ciudad de México, México.

## Conflict of Interest

The authors declare that the research was conducted in the absence of any commercial or financial relationships that could be construed as a potential conflict of interest.

## Publisher's Note

All claims expressed in this article are solely those of the authors and do not necessarily represent those of their affiliated organizations, or those of the publisher, the editors and the reviewers. Any product that may be evaluated in this article, or claim that may be made by its manufacturer, is not guaranteed or endorsed by the publisher.

## Abbreviations

BEE: basal energy expenditure
BF: body fat
BIA: bioelectrical impedance analysis
BMI: body mass index
BMISD: body mass index standard deviation
BSA: body surface area
CenI: centrality index
CNS: central nervous system
ConI: conicity index
DEXA: dual-energy X-ray absorptiometry
DSM
Diagnostic and Statistical Manual of Mental Disorders
FIO_2_: fractional inspiratory oxygen fraction
FFM, fat-free mass, FM: fat mass
G3: gonadal pubertal stage 3
Hb: hemoglobin
IC: indirect calorimetry
ICU: intensive care unit
IOM: Institute of Medicine
MUAMC: mid-upper arm muscle circumference
NHLBI, National Heart: Lung and Blood Institute
PBEE: basal energy expenditure
%BF: percentage body fat
PEEP: positive end-expiratory pressure
PIBW: percent ideal body weight
R: Pearson's correlation coefficient
*R* ^2^: coefficient of determination
REE: resting energy expenditure
RQ: respiratory quotient
SJS: Stevens-Johnson syndrome
TEE: total energy expenditure
TEN: toxic epidermal necrolysis
TPN: total parenteral nutrition
VCO_2_: carbon dioxide elimination
VO_2_: oxygen consumption
WAZ score: weight-for-age *Z-*score.
