# Supplementary material for: Resting Energy Expenditure Prediction Equations in the Pediatric Population: A Systematic Review
Source: Front Pediatr. 2021 Dec 6;9:795364. doi: 10.3389/fped.2021.795364 (PMC8685418; doi:10.3389/fped.2021.795364)
Supplement: Supplementary file 1 [file Table_1.docx]

**Table S1. Quality Assessment Tool for Observational Cohort and Cross-Sectional Studies.**

| **Criteria** | **Acar-Tek et al. 2017** | **Bertoli et al**  **.2020** | **Buchowski et al. 2002** | **Chan et al.**  **2009** | **Chu et al.**  **2019** | **Derumeaux-burel et al.2004** | **Dietz et al.**  **1991** | **FAO/OMS**  **1985** | **Goran et al.**  **1991** | **Harris Benedict 1918** | **Henry et al.**  **1999** | **Henry et al.**  **2005** | **IOM 2002** |
| --- | --- | --- | --- | --- | --- | --- | --- | --- | --- | --- | --- | --- | --- |
| 1. Was the research question or objective in this paper clearly stated? | Yes | Yes | Yes | Yes | No | Yes | No | No | Yes | Yes | Yes | Yes | No |
| 2. Was the study population clearly specified and defined? | Yes | Yes | Yes | Yes | Yes | Yes | No | Yes | Yes | Yes | Yes | Yes | Yes |
| 3. Was the participation rate of eligible persons at least 50%? | Yes | Yes | Yes | Yes | Yes | Yes | Yes | Yes | Yes | Yes | Yes | Yes | Yes |
| 4. Were all the subjects selected or recruited from the same or similar populations (including the same time period)? Were inclusion and exclusion criteria for being in the study prespecified and applied uniformly to all participants? | Yes | Yes | Yes | Yes | Yes | Yes | NR | NR | NR | No | No | Yes | No |
| 5. Was a sample size justification, power description, or variance and effect estimates provided? | No | No | Yes | No | No | No | No | No | No | No | No | No | No |
| 6. For the analyses in this paper, were the exposure(s) of interest measured prior to the outcome(s) being measured? | No | No | No | No | No | No | No | No | No | No | No | No | No |
| 7. Was the timeframe sufficient so that one could reasonably expect to see an association between exposure and outcome if it existed? | No | No | No | No | No | No | No | No | No | No | No | No | No |
| 8. For exposures that can vary in amount or level, did the study examine different levels of the exposure as related to the outcome (e.g., categories of exposure, or exposure measured as continuous variable)? | NA | NA | NA | NA | NA | NA | NA | NA | NA | NA | NA | NA | NA |
| 9. Were the exposure measures (independent variables) clearly defined, valid, reliable, and implemented consistently across all study participants? | Yes | Yes | Yes | Yes | Yes | Yes | No | NR | Yes | Yes | Yes | NR | Yes |
| 10. Was the exposure(s) assessed more than once over time? | NA | NA | NA | NA | NA | NA | NA | NA | NA | NA | NA | NA | NA |
| 11. Were the outcome measures (dependent variables) clearly defined, valid, reliable, and implemented consistently across all study participants? | Yes | Yes | Yes | Yes | Yes | Yes | Yes | NR | Yes | Yes | Yes | No | No |
| 12. Were the outcome assessors blinded to the exposure status of participants? | NA | NA | NA | NA | NA | NA | NA | NA | NA | NA | NA | NA | NA |
| 13. Was loss to follow-up after baseline 20% or less? | NA | NA | NA | NA | NA | NA | NA | NA | NA | NA | NA | NA | NA |
| 14. Were key potential confounding variables measured and adjusted statistically for their impact on the relationship between exposure(s) and outcome(s)? | Yes | Yes | Yes | Yes | Yes | Yes | Yes | Yes | Yes | Yes | Yes | Yes | Yes |
| **Quality Rating (Good, Fair, or Poor)** | Good | Good | Good | Good | Fair | Good | Poor | Poor | Fair | Fair | Fair | Fair | Poor |
| **Comments (If POOR, please state why):** |  |  |  |  |  |  | The objective and the population were not well defined | The objective and the sample size were not well defined |  |  |  |  | The objective and the population were not well defined |

Rater #1 Initials: JFS

Rater #2 Initials: LGS

NA, not applicable and NR, not reported. The Quality Rating of each study was rated as Good, Fair or Poor.

**Table S1. Quality Assessment Tool for Observational Cohort and Cross-Sectional Studies. (Continue 2)**

| **Criteria** | **Jhang et al.**  **2020** | **Kaneko et al.**  **2013** | **Lawrence et al. 2009** | **Lazzer et al. 2006** | **Lazzer et al. 2010** | **Lazezr et al. 2014** | **Maffeis et al. 1993** | **Mayes et al. 1996** | **Mayes et al. 2008** | **McDuffie et al. 2004** | **Mehta et al. 2015** | **Meyer et al. 2012** | **Molnár et al. 1995** |
| --- | --- | --- | --- | --- | --- | --- | --- | --- | --- | --- | --- | --- | --- |
| 1. Was the research question or objective in this paper clearly stated? | Yes | Yes | Yes | Yes | No | Yes | Yes | No | Yes | No | Yes | Yes | Yes |
| 2. Was the study population clearly specified and defined? | Yes | Yes | Yes | Yes | Yes | Yes | Yes | Yes | Yes | Yes | Yes | Yes | Yes |
| 3. Was the participation rate of eligible persons at least 50%? | Yes | Yes | Yes | No | Yes | Yes | Yes | Yes | Yes | Yes | Yes | Yes | Yes |
| 4. Were all the subjects selected or recruited from the same or similar populations (including the same time period)? Were inclusion and exclusion criteria for being in the study prespecified and applied uniformly to all participants? | Yes | NR | NR | Yes | Yes | Yes | Yes | Yes | Yes | Yes | Yes | Yes | Yes |
| 5. Was a sample size justification, power description, or variance and effect estimates provided? | No | No | Yes | No | No | No | No | No | No | No | No | No | No |
| 6. For the analyses in this paper, were the exposure(s) of interest measured prior to the outcome(s) being measured? | No | No | No | No | No | No | No | No | No | No | No | No | No |
| 7. Was the timeframe sufficient so that one could reasonably expect to see an association between exposure and outcome if it existed? | No | No | No | No | No | No | No | No | No | No | No | No | No |
| 8. For exposures that can vary in amount or level, did the study examine different levels of the exposure as related to the outcome (e.g., categories of exposure, or exposure measured as continuous variable)? | NA | NA | NA | NA | NA | NA | NA | NA | NA | NA | NA | NA | NA |
| 9. Were the exposure measures (independent variables) clearly defined, valid, reliable, and implemented consistently across all study participants? | Yes | Yes | Yes | Yes | Yes | Yes | Yes | No | No | Yes | Yes | Yes | Yes |
| 10. Was the exposure(s) assessed more than once over time? | NA | NA | NA | NA | NA | NA | NA | NA | NA | NA | NA | NA | NA |
| 11. Were the outcome measures (dependent variables) clearly defined, valid, reliable, and implemented consistently across all study participants? | Yes | Yes | Yes | Yes | Yes | Yes | Yes | No | No | Yes | Yes | Yes | Yes |
| 12. Were the outcome assessors blinded to the exposure status of participants? | NA | NA | NA | NA | NA | NA | NA | NA | NA | NA | NA | NA | NA |
| 13. Was loss to follow-up after baseline 20% or less? | NA | NA | NA | NA | NA | NA | NA | NA | NA | NA | NA | NA | NA |
| 14. Were key potential confounding variables measured and adjusted statistically for their impact on the relationship between exposure(s) and outcome(s)? | Yes | Yes | Yes | Yes | Yes | Yes | Yes | Yes | Yes | Yes | No | Yes | Yes |
| **Quality Rating (Good, Fair, or Poor)** | Good | Fair | Good | Fair | Fair | Good | Good | Poor | Fair | Fair | Fair | Good | Good |
| **Comments (If POOR, please state why):** |  |  |  |  |  |  |  | The objective and sample size were not well defined |  |  |  |  |  |

Rater #1 Initials: JFS

Rater #2 Initials: LGS

NA, not applicable and NR, not reported. The Quality Rating of each study was rated as Good, Fair or Poor.

**Table S1. Quality Assessment Tool for Observational Cohort and Cross-Sectional Studies. (Continue 3)**

| **Criteria** | **Moukarzel et al. 2003** | **Müller et al. 2004** | **Pierro et al. 1994** | **Salas et al. 1990** | **Scalfi et al. 2001** | **Schmelzle et al. 2004** | **Schofield et al. 1985** | **Tounian et al. 1993** | **Tverskaya et al. 1998** | **Uemura et al. 2011** | **White et al. 2000** | **Williams et al. 2002** | **Zhang et al. 2018** |
| --- | --- | --- | --- | --- | --- | --- | --- | --- | --- | --- | --- | --- | --- |
| 1. Was the research question or objective in this paper clearly stated? | Yes | Yes | Yes | Yes | Yes | Yes | Yes | No | Yes | No | Yes | Yes | No |
| 2. Was the study population clearly specified and defined? | No | Yes | No | No | Yes | Yes | Yes | No | Yes | Yes | Yes | Yes | Yes |
| 3. Was the participation rate of eligible persons at least 50%? | Yes | CD | Yes | Yes | Yes | Yes | Yes | Yes | Yes | Yes | Yes | Yes | Yes |
| 4. Were all the subjects selected or recruited from the same or similar populations (including the same time period)? Were inclusion and exclusion criteria for being in the study prespecified and applied uniformly to all participants? | No | Yes | Yes | Yes | Yes | Yes | No | NR | NR | Yes | No | No | Yes |
| 5. Was a sample size justification, power description, or variance and effect estimates provided? | No | No | No | No | No | No | No | No | No | No | No | No | No |
| 6. For the analyses in this paper, were the exposure(s) of interest measured prior to the outcome(s) being measured? | No | No | No | No | No | No | No | No | No | No | No | No | No |
| 7. Was the timeframe sufficient so that one could reasonably expect to see an association between exposure and outcome if it existed? | No | No | No | No | No | No | No | No | No | No | No | No | No |
| 8. For exposures that can vary in amount or level, did the study examine different levels of the exposure as related to the outcome (e.g., categories of exposure, or exposure measured as continuous variable)? | NA | NA | NA | NA | NA | NA | NA | NA | NA | NA | NA | NA | NA |
| 9. Were the exposure measures (independent variables) clearly defined, valid, reliable, and implemented consistently across all study participants? | Yes | Yes | Yes | Yes | Yes | Yes | No | Yes | Yes | Yes | Yes | Yes | Yes |
| 10. Was the exposure(s) assessed more than once over time? | NA | NA | NA | NA | NA | NA | NA | NA | NA | NA | NA | NA | NA |
| 11. Were the outcome measures (dependent variables) clearly defined, valid, reliable, and implemented consistently across all study participants? | Yes | Yes | Yes | Yes | Yes | Yes | Yes | Yes | Yes | Yes | Yes | Yes | Yes |
| 12. Were the outcome assessors blinded to the exposure status of participants? | NA | NA | NA | NA | NA | NA | NA | NA | NA | NA | NA | NA | NA |
| 13. Was loss to follow-up after baseline 20% or less? | NA | NA | NA | NA | NA | NA | NA | NA | NA | NA | NA | NA | NA |
| 14. Were key potential confounding variables measured and adjusted statistically for their impact on the relationship between exposure(s) and outcome(s)? | Yes | Yes | Yes | Yes | Yes | Yes | Yes | Yes | Yes | Yes | Yes | Yes | Yes |
| **Quality Rating (Good, Fair, or Poor)** | Fair | Fair | Fair | Fair | Good | Good | Fair | Poor | Fair | Fair | Fair | Fair | Fair |
| **Comments (If POOR, please state why):** |  |  |  |  |  |  |  |  |  |  |  |  |  |

Rater #1 Initials: JFS

Rater #2 Initials: LGS

NA, not applicable and NR, not reported. The Quality Rating of each study was rated as Good, Fair or Poor.
