# Supplementary figures and images for: Resting Energy Expenditure Prediction Equations in the Pediatric Population: A Systematic Review
Source: Front Pediatr. 2021 Dec 6;9:795364. doi: 10.3389/fped.2021.795364 (PMC8685418; doi:10.3389/fped.2021.795364)

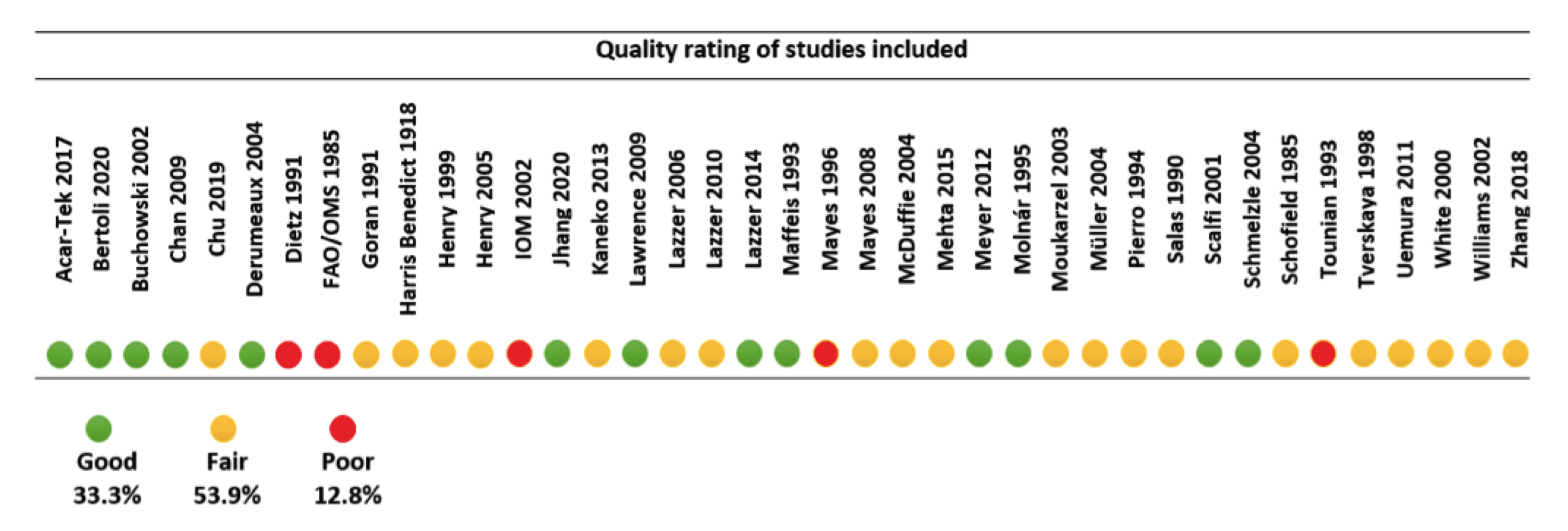

Supplement: Supplementary file 2 [file Image_1.TIFF]
